# Potential efficacy of dopaminergic antidepressants in treatment resistant anergic-anhedonic depression results of the chronic anergic-anhedonic depression open trial – CADOT

**DOI:** 10.3389/fpsyt.2023.1194090

**Published:** 2023-09-27

**Authors:** Ludovic Christophe Dormegny-Jeanjean, Clément de Billy, Olivier Mainberger, Sébastien Weibel, Benoit Schorr, Alexandre Obrecht, Lionel Landré, Fabrice Berna, Jean-Baptiste Causin, Frederic Blanc, Vlad Danila, Mihaela Tomsa, Geraldine Pfleger, Camille Meyer, Ilia Humbert, Hervé Javelot, Guillaume Meyer, Gilles Bertschy, Jack Rene Foucher

**Affiliations:** ^1^Treatment resistant depression expert center of Alsace (CEDRA), Strasbourg-Rouffach-Erstein-Brumath, Rouffach, France; ^2^Non-Invasive neuroModulation Center of Strasbourg (CEMNIS), University Hospital of Strasbourg, Strasbourg, France; ^3^CNRS UMR 7357 iCube, neurophysiology, FMTS, University of Strasbourg, Strasbourg, France; ^4^Department of Psychiatry and Mental Health – University Hospital of Strasbourg, University of Strasbourg, Strasbourg, France; ^5^INSERM UMR 1114, Physiopathology and Cognitive Psychopathology of Schizophrenia, University of Strasbourg, Strasbourg, France; ^6^Geriatrics Department and Expert Center for Neurocognitive Disorders, University Hospital of Strasbourg, Strasbourg, France; ^7^Department of Psychiatry “Pole 8/9”, Rouffach Psychiatric Hospital, Rouffach, France; ^8^Department of Integrated Psychiatric Care, Centre Hospitalier d’Erstein, Erstein, France; ^9^Expert center in Psychopharmacology, Etablissement public de santé Alsace nord (EPSAN), Bischwiller, France; ^10^Department of Psychopharmacology, Centre Hospitalier d’Erstein, Lingolsheim, France

**Keywords:** treatment resistant depression, anergia, anhedonia, apathy, monoamine oxidase inhibitors, dopamine D2 receptor agonists, atypical depression, hypodopaminergic mesencephalic syndrome

## Abstract

**Introduction:**

Among treatment-resistant depression (TRD), we identified anergic-anhedonic clinical presentations (TRAD) as putatively responsive to pro-dopaminergic strategies. Based on the literature, non-selective monoamine oxidase inhibitors (MAOI) and dopamine D2 receptor agonists (D2RAG) were sequentially introduced, frequently under the coverage of a mood stabilizer. This two-step therapeutic strategy will be referred to as the Dopaminergic Antidepressant Therapy Algorithm (DATA). We describe the short and long-term outcomes of TRAD managed according to DATA guidelines.

**Method:**

Out of 52 outpatients with TRAD treated with DATA in a single expert center, 48 were included in the analysis [severity – QIDS (Quick Inventory of Depressive Symptomatology) = 16 ± 3; episode duration = 4.1 ± 2.7 years; Thase and Rush resistance stage = 2.9 ± 0.6; functioning – GAF (Global Assessment of Functioning) = 41 ± 8]. These were followed-up for a median (1st – 3rd quartile) of 4 (1–9) months before being prescribed the first dopaminergic treatment and remitters were followed up 21 (11–33) months after remission.

**Results:**

At the end of DATA step 1, 25 patients were in remission (QIDS <6; 52% [38–66%]). After DATA step 2, 37 patients were in remission (77% [65–89%]) to whom 5 patients with a QIDS score = 6 could be added (88% [78–97%]). Many of these patients felt subjectively remitted (GAF = 74 ± 10). There was a significant benefit to combining MAOI with D2RAG which was maintained for at least 18 months in 30 patients (79% [62–95%]).

**Conclusion:**

These results support TRAD sensitivity to pro-dopaminergic interventions. However, some clinical heterogeneities remain in our sample and suggest some improvement in the description of dopamine-sensitive form(s).

## 1. Introduction

Until recently, depression was mostly considered to be a single entity, thus legitimizing one-size-fits-all therapeutic guidelines (1–3). This view, based on an atheoretical approach of consensus criteria, has some limitations. These are epitomized by the Sequenced Treatment Alternatives to Relieve Depression (STAR*D) trial showing the virtual absence of superiority of one therapeutic strategy over another, resulting in 33% of non-remitters despite four successive lines of treatment (4), a proportion which grows up to 54% when dropouts are considered, i.e., when STAR*D is analyzed as a whole from an intention-to-treat perspective (ITT) (5).

Yet the reification of ‘depressive disorder’ as equivalent to a disease or a syndrome underpinned by a common pathophysiology is more of a belief than a factual statement (6, 7). Depression is more likely a broad defined condition encompassing multiple syndromes or diseases whose different causes make them more responsive to specific interventions. Considering heterogeneities in mood disorders aims to avoid the trial-and-error approach promoted by current guidelines and to focus on the most appropriate treatments, i.e., precision medicine (8, 9). The analogy between dopamine withdrawal apathy and anergic-anhedonic depression led us to reinterpret the latter as a mesolimbic hypodopaminergic syndrome likely to respond to dopamine-enhancing strategies.

In the 2010s, movement disorders specialists described the emergence of apathy after surgery for Parkinson’s disease. Five months after subthalamic nucleus deep brain stimulation, half of the patients developed a loss of motivation, a difficulty in initiating goal-directed cognitions and actions by themselves coming together with anhedonia, which could develop to emotional numbness and depression (10). Accumulating evidence suggests that this symptomatology is the result of mesolimbic hypodopaminergia: (i) apathy is unmasked by the rapid reduction of dopaminergic drugs allowed by the dramatic improvement of motor symptoms, (ii) it is reversed by dopamine D2 receptor agonists (D2RAG) (10, 11), and (iii) dopaminergic denervation was found in the ventral striatum, amygdala, and medial and lateral orbito-frontal cortices of apathetic patients (12).

Dopamine withdrawal apathy was a reminder of some clinical pictures of depression, not only in their clinical presentation but also their pharmacological reactivity. Soon after its discovery, iproniazid, the first non-selective irreversible monoamine oxidase inhibitor (MAOI), was reported to be especially effective on ‘inert psychasthenic-anhedonic reactions’ (13). Ten years later, the Pittsburgh group showed ‘anergic depression’, characterized by a loss of initiative experienced as a volitional inhibition (anergia), anhedonia, and reversed vegetative symptoms (hypersomnia and hyperphagia), to be selectively responsive to tranylcypromine (TCP) – another MAOI (14–16). Another decade later, the Columbia group redefined ‘atypical depression’ as emotional hyper-reactivity, leaden paralysis, and reversed neurovegetative symptoms, and showed its responsivity to phenelzine – again a MAOI (17, 18). Anergic and atypical depressions were originally thought to be of different natures: a clinical picture of manic-depressive illness for the former (i.e., endogenous) vs. non-endogenous (i.e., neurotic) depression frequently developing on personality disorders for the latter (19). Yet they shared features like fatigue and reversed neurovegetative symptoms and responded to MAOI. In the following, this designation refers only to irreversible and non-selective drugs. By inhibiting both A and B isoforms, MAOI is one of the very few classes of pro-dopaminergic antidepressants (20).

Analogical reasoning with the mesolimbic hypodopaminergic syndrome led us to treat three patients with chronic and severe treatment-resistant depression (TRD) presenting prominent anergic-anhedonic features with MAOI and D2 receptor agonists (D2RAG). The effect was dramatic, which led us to formalize the ‘Dopaminergic Antidepressant Therapy Algorithm’ (DATA) for treatment-resistant anergic-anhedonic depression (TRAD).

But in line with the seminal report of the Pittsburgh group, one patient was already diagnosed as bipolar (and treated with lithium) whereas the two ‘apparently unipolar’ patients switched to mania (14, 19). Like the Pittsburgh group, this led us to speculate that TRAD belongs to the bipolar spectrum and we designed diagnostic criteria and therapeutic guidelines accordingly, i.e., DATA initially emphasized the preventive introduction of antimanic mood-stabilizers (see methods). The Chronic Anergic-anhedonic Depression Open Trial (CADOT) was designed to evaluate the effectiveness of DATA guidelines in the routine management of TRAD (efficacy and side-effects). The following article reports the short and long-term outcomes and explores the predictors of remission.

## 2. Materials and methods

### 2.1. Definitions

#### 2.1.1. Defining the target population: TRAD

Based on first results, we redefined anergic-anhedonic depression in 2012 (last revised in 2013). Mesolimbic hypodopaminergic syndrome was used as guidance, but we included features that could help anticipate unrevealed bipolar depression (21–23) and remained open to ‘atypical’/‘neurotic’ forms (19, 24). Treatment-resistant anergic-anhedonic depression (TRAD) hereafter refers to the absence of response to two lines of antidepressants in anergic-anhedonic patients fulfilling the criteria listed in Table 1.

**Table 1:** Diagnostic criteria of anergic-anhedonic depression used in the study.

Diagnostic criteria for anergic-anhedonic depression
Rank A: all of the following must be fulfilled
Anergia: loss self-generated action and thought, volitional inhibition, poor motivation. Anhedonia and/or emotional numbness. Significant impact on personal or professional life (a decrease ≥20 pts. in GAF score and/or a GAF score ≤50) and significant duration ≥3 months.
Rank B: one or more of the following must be present
Sadness secondary to self-criticism or guilt in relation with the poverty of action and emotion^¤^. Atypical depression or at least one reversed neurovegetative symptom (one or more): Overeating and/or weight gain (≥10% of initial weight), Hypersomnia (≥10 h of sleep per day or increase ≥+2 h). ±heavy sensation in the limbs and/or interpersonal rejection reactivity. WKL-MDI (one or more): Mixed or incomplete states (or poles)*. Mood reactivity and/or polymorphic and/or fluctuating clinical manifestations. Psychiatric history with ≥1 manic/hypomanic + ≥1 depressive episodes. First degree relative suffering of bipolar I disorder or WKL-MDI.
Exclusion criteria: symptoms are not attributable to
Moderate or absence of thought inhibition and dysexecutive syndrome^†^. Current drug abuse or withdrawal^‡^. Medical condition (including neurodegenerative diseases).

Translation from French criteria first posted on 10 Dec 2012, last updated on 13 Oct 2013 (French original version, 2013’s English translation). GAF, Global Assessment of Functioning; WKL-MDI, Wernicke-Kleist-Leonhard’s definition of manic-depressive illness. *“Mixed states are defined as the co-occurrence of both the manic and depressive pole among the different domains: affect, thought, and psychomotricity. […] Incomplete states are an extension of the former concept, meaning that aside from being excited and inhibited, a single domain can also be completely unaffected” (22); ^¤^The lack of motivation, the difficulty in initiating action, or the poverty of emotion leading to the feeling of failure to meet commitments or obligations, to self-criticism, to withdraw from social or family life, or to guilt for not feeling affectionate emotions towards loved ones. ^♱^Poverty of action is not primarily explained by difficulties in problem solving, i.e., orienting and maintaining attention, and planning and monitoring actions, as seen in dementia or severe obsessive–compulsive disorder (mental overload and procrastination). ^‡^Opioid withdrawal syndrome, especially during or after tapering of opioid substitution therapy, responds to reintroduction, whereas D2RAG may promote addictive behavior.

#### 2.1.2. DATA: a two-step dopaminergic antidepressant therapy algorithm

**Figure 1:**
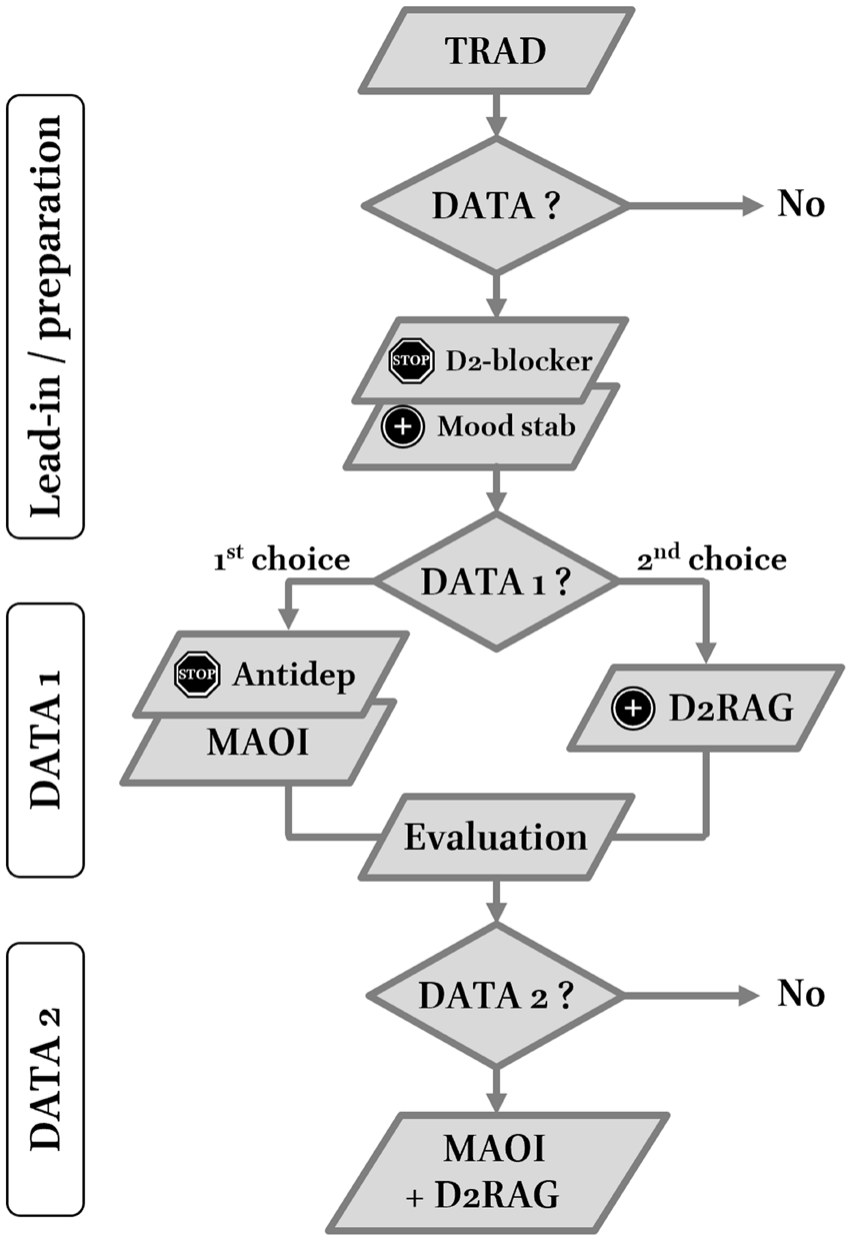
Two-step Dopaminergic Antidepressant Therapy Algorithm (DATA) for treatment resistant anergic-anhedonic depression (TRAD). Lead-in/preparation period: patients who accepted were first proposed to stop or reduce D2 receptor blockers and to introduce a mood stabilizer (preferably lithium). DATA1 (first step): patients were advised to switch to a non-selective (irreversible) monoamine oxidase inhibitor (MAOI – first choice). The add-on of dopamine D2 receptor agonist (D2RAG) to current antidepressant (classical) was presented as the second choice. DATA2 (second step): all non-remitters or patients who did not feel like before were proposed to combine MAOI and D2RAG. At each step, the prescriber was allowed to change molecules within the same class and adapt titration speed to optimize tolerance. Evaluations with QIDS were performed after sufficient stability to allow management decisions to maintain, stop, or combine treatments.

The clinical characteristics of TRAD were supposed to be predictive of a good response to a dopaminergic strategy, i.e., DATA (Figure 1). Not included in the counting of steps is the preparatory phase. It was first recommended to (i) exclude contraindications for MAOI and/or D2RAG, (ii) remove medications that may impede dopamine transmission, e.g., foremost to stop D2-blockers, and (iii) initiate an antimanic mood-stabilizer. Lithium was initially strongly recommended. Over time, though lithium still was proposed as the first choice, lamotrigine was considered in the absence of previous manic or hypomanic episodes or bipolar relatives.

DATA was presented as a two-step dopaminergic approach aiming at full remission. The first step (DATA1) consisted of the introduction of a first dopaminergic class. The decision was discussed with the patient but a switch to MAOI was presented as the first choice relative to the add-on of D2RAG to the current antidepressant (preferentially a serotonin–norepinephrine reuptake inhibitor or SNRI). In 2013, iproniazid was the only MAOI available in France but was discontinued in 2015. By then, TCP was proposed as the first-choice because of its tolerance profile and efficiency in the Pittsburgh studies (15, 16, 19). Phenelzine was only considered as a first line MAOI in cases of prominent anxiety (25). Transdermal selegiline was mostly a second choice. Titration was adapted to tolerance but generally performed on a weekly basis up to the minimum effectiveness. The prescriber waited about four weeks to assess effectiveness, during which side effects were managed, before eventually increasing doses up to remission or the maximally tolerated dose.

D2RAG were considered in add-on, i.e., combined with an ongoing antidepressant (preferentially an SNRI). Pramipexole (PPX) was the first-choice D2RAG, initially with a three-doses-per-day scheme (as in Parkinson’s disease). In 2018, concerns about D2RAG-induced heart failure and reports of efficacy when delivered once a day led to the recommendation of a single dose in the evenings (26).

In the absence of subjective remission after dose optimization, side effect management, and the sufficient duration (≥6 weeks) of DATA1, the patient was proposed to enter DATA2, i.e., the combination of MAOI with D2RAG.

### 2.2. Chronic anergic-anhedonic depression open trial – CADOT design

CADOT was designed as a monocentric, prospective observational mirror-image study. It started in 2013 after proof-of-concept in a few patients and ended in June 2021. As CADOT aimed to evaluate DATA as a guideline for the treatment of chronic anergic-anhedonic depression in routine care, it was not formerly registered and only announced on a dedicated web page (http://www.cercle-d-excellence-psy.org/bienvenue/nouvellestypenews/?tx_ttnews%5btt_news%5d=19&cHash=ff1592fbfcb708577d02298d85ed4297).

All patients coming to the TRD expert center were screened for TRAD. At the time, this quaternary care facility of the University Hospitals of Strasbourg could only provide outpatient care. Out of the exclusion criteria mentioned in Table 1, patients were screened for contraindication to MAOI and/or D2RAG before being proposed DATA (27). Patients’ characteristics and assessments were collected prospectively except the duration of the remission and tolerance data which were retrospectively extracted from patients’ files. Treatment resistance was assessed using the Thase and Rush staging model (28). Symptom severity was evaluated by the Quick Inventory of Depressive Symptomatology clinician-rated version (QIDS), which is more sensitive to TRAD-specific ‘atypical’ features (hyperphagia and hypersomnia) (29). The QIDS was first assessed at the visit when the patient was prescribed DATA1 treatment, i.e., MAOI or D2AGO (excluding the preparatory phase). Interim and final evaluations were not performed at a specific time after initiation but when, after adapting the treatment at best, the physician felt that the patient was remitted or, if non-remitted, that no further improvement could be achieved. Finally, the Global Assessment of Functioning Scale (GAF) was only administrated at the beginning and at the end of the trial (with no interim evaluation) (30).

Since different molecules could be used, MAOI doses were converted into tranylcypromine equivalent doses (TCP-eq) (31, 32) and D2RAG into pramipexole equivalent doses (PPX-eq) (33, 34).

For all patients, the diagnosis was rediscussed with the physician in charge and management difficulties, e.g., deviation from the guidelines, were retrospectively examined.

CADOT received approval from the ethics committee of the University Hospitals of Strasbourg (approval reference no. CE-2023-31). All patients were contacted and consented to the use of their data.

### 2.3. Analysis plan

#### 2.3.1. Primary outcome and analysis

The primary outcome was the proportion of remitters (Rm) defined by a QIDS score <6 (29).

The trial was analyzed from an ITT perspective. In accordance with the mirror image design, the only planned comparison was the proportion of Rm after DATA1 and after DATA2, i.e., the added value of the combination relative to a single drug (Odds ratio; *χ*^2^-test, *α* = 0.05).

#### 2.3.2. Secondary outcomes and analyses

The proportion of responders (Rs) was computed to compare our results with the literature. Patients were considered as Rs if they were Rm or had their QIDS score reduced by at least 50%.

The absence of response could be related to resistance despite adequate trial, but also to early dropout, e.g., due to side effects. An adequate trial was defined as treatment maintained for at least 6 weeks at the minimum effective dose (MAOI ≥30 mg/d TCP-eq; D2RAG > 1 mg/d PPX-eq).

The time to remission was calculated from the first prescription to the first visit showing a remission that subsequently proved to be stable (≥6 weeks). In addition to the time of effectiveness, the time to Rm includes the progressive dose increases and the management of side effects which sometimes led to a change of molecule.

The time to trial completion was calculated from the first prescription to the management decision visit, i.e., pursuing the treatment, moving to DATA2, or quitting. For Rm, the time to the end of the trial equaled the time to remission. In non-remitters (nRm), the time to trial completion could be shorter if the patient quit because of side effects or longer because it took more time to conclude that the treatment had been best adapted.

Considering the paucity of missing data, no data imputation was performed. The Kaplan–Meier method was used to generate the right-censored survival curves of Rm and Rs. Post-hoc exploratory Student, Wilcoxon, or *χ*^2^-test were performed on groups characteristics for continuous, non-Gaussian, and categorial data, respectively. Considering their exploratory purpose, no adjustment was made for multiple comparisons (*α* = 0.05, two-sided). Effect size was converted to standardized distance (‘*d*’ – see Supplementary material).

#### 2.3.3. Conventions

Proportions are given in percentages, sometimes followed by 95% confidence intervals [in square brackets], computed using the normal approximation to the binomial distribution. Continuous values are summarized by their mean ± standard deviation except time to remission and time to trial completion which had left fat-tailed distributions. These were summarized by their median and their interquartile range in round brackets (Q1 – Q3).

## 3. Results

### 3.1. Patients

**Figure 2:**
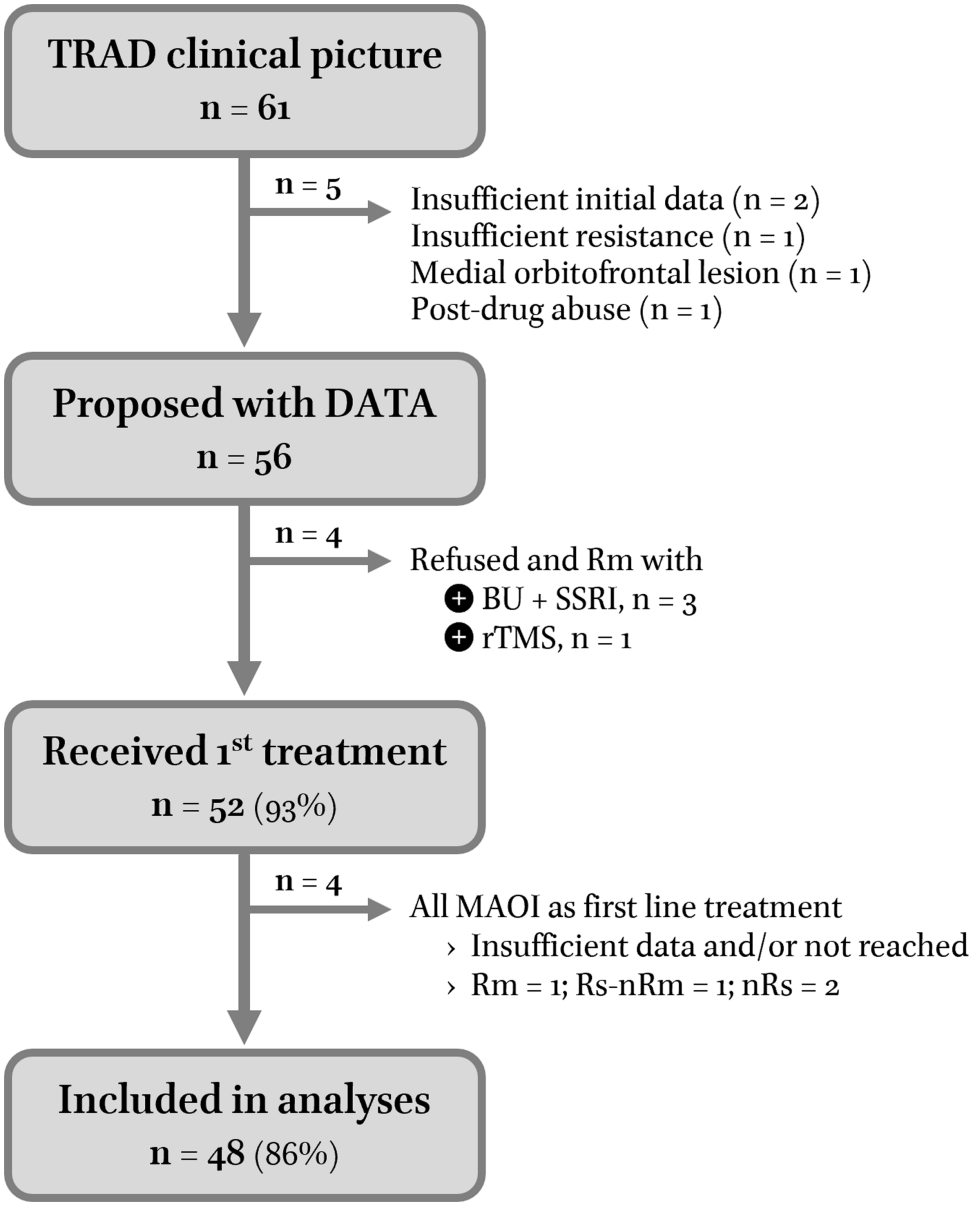
Flow chart of Chronic Anergic-anhedonic Depression Open Trial (CADOT). In total, 56 out of 61 TRAD outpatients presenting to Strasbourg’s TRD reference center between 2013 and mid-2021 were proposed to be treated along DATA guidelines (none had contraindications to dopaminergic treatment). Of the four patients who wanted to try another option and remitted (Rm), three benefited from the combination of bupropion to an SSRI which is already a dopaminergic strategy. Because bupropion is not covered by the French health insurance system, it was not considered in this analysis. Only a few patients who could afford it had a bupropion trial before entering DATA. It should be noted that no patients responded to mood stabilizers alone. However, antidepressant washout periods longer than 8 weeks (mood stabilizers alone) were systematically offered to bipolar patients only until 2017. The lack of success led us to be less insistent thereafter.

In total, 56 of 61 outpatients diagnosed with TRAD were proposed with MAOI as the first line treatment. Four chose and responded to other therapeutic options: three bupropion-SNRI combinations and one individualized rTMS (7%) (35). Of the 52 subjects who entered DATA1, four (8%) received MAOI but could not be included in the analysis because of insufficient data: one Rm, one Rs, and two non-responders (nRs: i.e., implicitly non-remitters nRm). None received the combo, i.e., DATA2 (see Figure 2 for the flow-chart of inclusions).

The baseline demographic and clinical characteristics of the 48 (93%) patients who were included are reported in Table 2, Section 2a. TRAD was severe (QIDS = 16 ± 3) and heavily disabling (GAF = 41 ± 8). The current episodes were resistant to treatments (≈stage III, i.e., having failed at least three trials with different classes including tricyclic antidepressants) and of long duration (3.3 ± 2.4 years). Thirty patients (63%) were chronically depressed (i.e., >2 years).

**Table 2:** Populations’ baseline characteristics.

Population characteristics	2.a. General	2.b. DATA1			2.c. DATA2		
		MAOI	D2RAG	*p*	No	Yes	*p*
Population (*N*)	48	38 (79%)	10 (21%)		32 (67%)	16 (33%)	
Female/Male (% female)	22/26 (46%)	21/17 (55%)	1/9 (10%)	**0.011**	13/19 (41%)	9/7 (56%)	–
Age (years)	59 ± 12	58 ± 13	63 ± 8	–	58 ± 11	60 ± 13	–
Age at first episode (years)	42 ± 16	41 ± 17	45 ± 17	–	40 ± 15	44 ± 20	–
Number of previous episodes	3 ± 3	3 ± 3	2 ± 2	–	3 ± 3	3 ± 3	–
Bipolar disorder*	14 (29%)	12 (32%)	2 (20%)	–	8 (25%)	6 (38%)	–
Affected first degree relative	30 (63%)	22 (58%)	8 (80%)	–	21 (66%)	9 (56%)	–
Lead-in period (weeks)	19 (5–37)	15 (2–37)	8 (2–23)	–	19 (3–30)	6 (1–34)	–
Current episode resistance							
Duration of the episode (years)^¤^	4.1 ± 2.7	4.1 ± 2.5	4.2 ± 3.8	–	4.4 ± 2.8	3.6 ± 2.4	–
Thase and Rush resistance	2.9 ± 0.6	3.1 ± 0.7	2.2 ± 0.7	**0.01**	2.6 ± 0.6	3.5 ± 1	**0.008**
›SSRI	46 (96%)	37 (97%)	9 (90%)	–	31 (97%)	15 (94%)	–
›SNRI	42 (88%)	34 (89%)	8 (80%)	–	26 (81%)	16 (100%)	*0.064*
›Tricyclics antidepressants	31 (65%)	25 (66%)	6 (60%)	–	21 (66%)	10 (63%)	–
›Antipsychotic	22 (46%)	19 (50%)	3 (30%)	–	11 (34%)	11 (69%)	**0.024**
›Combination or augmentation	37 (77%)	31 (82%)	6 (60%)	–	23 (72%)	14 (88%)	–
›rTMS	4 (8%)	3 (8%)	1 (10%)	–	1 (3%)	3 (19%)	*0.065*
›ECT	6 (13%)	6 (16%)	0 (0%)	–	1 (3%)	5 (31%)	**0.005**
Clinical features at baseline							
QIDS at baseline	16.4 ± 3.4	16.2 ± 3.4	17.1 ± 3.2	–	15.4 ± 3.2	17.8 ± 3.3	*0.057*
GAF at baseline	41 ± 8	40 ± 8	42 ± 9	–	42 ± 8	39 ± 9	–
›Anxiety	40 (83%)	31 (82%)	9 (90%)	–	27 (84%)	13 (81%)	–
›Psychotic features	8 (17%)	4 (11%)	4 (40%)	**0.026**	4 (13%)	4 (25%)	–
›Mixed or incomplete states^†^	35 (73%)	26 (68%)	9 (90%)	–	23 (72%)	12 (75%)	–
›Mood reactivity/fluctuations^†^	42 (88%)	34 (89%)	8 (80%)	–	29 (91%)	13 (81%)	–
›Atypical criteria*	13 (27%)	11 (29%)	2 (20%)	–	11 (34%)	2 (13%)	–
›Increased appetite	8 (17%)	7 (18%)	1 (10%)	–	5 (16%)	3 (19%)	–
›Increased weight (≥10%)	12 (25%)	9 (24%)	3 (30%)	–	7 (22%)	5 (31%)	–
›Hypersomnia	20 (42%)	15 (39%)	5 (50%)	–	11 (34%)	9 (56%)	–

QIDS, Quick Inventory of Depressive Symptoms, 16 items – clinician rated version; GAF, Global Assessment of Functioning; SSRI, selective serotonin reuptake inhibitors; SNRI, serotonin and norepinephrine reuptake inhibitors; rTMS, repetitive transcranial magnetic stimulation; ECT, electro-convulsive therapy. Means are expressed as “mean (±standard deviation)”; medians are expressed as “median (1st–3rd quartile).” *According to DSM-5 criteria (36); ¤Corresponds to the duration of the episode at the time of the first contact with Strasbourg’s expert center on TRD plus the lead-in period, i.e., until the first DATA1 treatment. ^†^The concepts of mixed or incomplete states and mood reactivity or fluctuation are defined according to Karl Leonhard (22). For sections 2b and 2c, the *p*-values of group-comparisons are in bold when the difference is significant (*α* = 0.05, bilateral, uncorrected for multiple testing), or in gray italic when only a trend (threshold = 0.1, bilateral, uncorrected for multiple testing). Effect sizes are provided in the Supplementary material.

### 3.2. Study process

**Figure 3:**
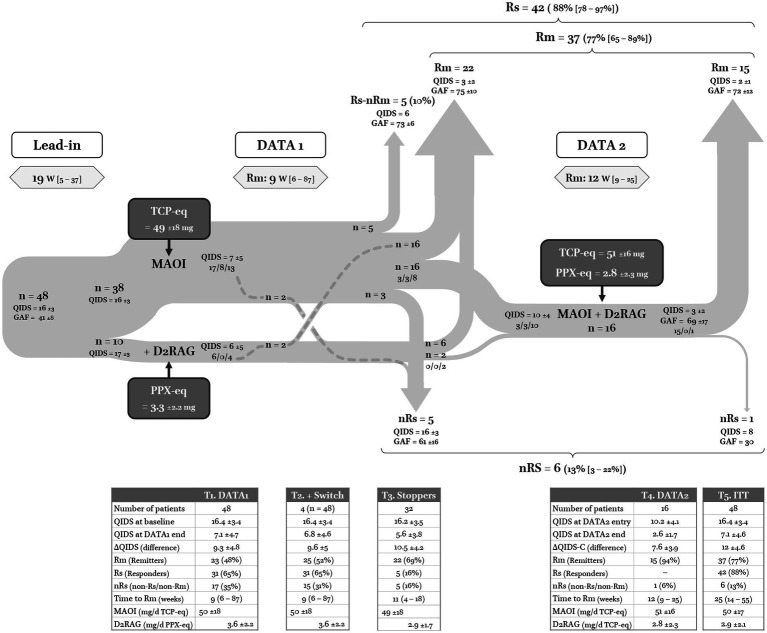
Flow chart of DATA. The three numbers separated by split lines corresponds to remitters (Rm), responders but non-remitters (Rs-nRm), and non-responders (nRs). T1 (Table 1): outcomes of the first dopaminergic trial. TCP-eq: tranylcypromine equivalent doses for MAOI; PPX-eq: pramipexole equivalent doses for D2RAG. T2: same as T1 but adding the four patients who switched from MAOI to D2RAG or vice versa. T3: outcomes for all patients who stopped after DATA1 (only one dopaminergic class). T4: outcomes of patients who entered DATA2. T5: outcomes of all patients who had one prescription according to DATA guidelines. ITT: intention-to-treat analysis.

The flow chart of the study is reported in Figure 3. The long preparation/lead-in period of 19 [5–37] weeks corresponds to the time it took to get patient approval of the DATA as a treatment scheme (meanwhile other treatments could be tried), to introduce a mood stabilizer, and to stop incompatible treatments, i.e., D2RAG and other antidepressants if MAOI was accepted as the first step.

#### 3.2.1. Data 1: MAOI or add-on of D2RAG

In line with DATA1, 38 (79%) patients were treated with MAOI (TCP-eq = 49 ± 18 mg) and 10 (21%) preferred starting with D2RAG (PPX = 3.3 ± 2.2 mg – Table 2, Section 2b). There was only one female in the D2RAG group, whereas the MAOI group was more equilibrated (*p* = 0.01). The MAOI group was more likely to suffer from psychotic depression (*p* < 0.05) and had a higher grade of resistance (*p* = 0.004, see Table 2, Section 2b).

Nearly half of the sample participants remitted, i.e., 23 patients were Rm (48%), 8 (17%) were Rs (but nRm), and 17 (35%) were nRs (Figure 3, T1). The time to remission was 9 [6–87] weeks and time to trial completion was 11 [7–22] weeks. There was no difference in the outcome between the MAOI and D2RAG groups.

#### 3.2.2. Stoppers and switchers

Four nRs did not proceed to DATA2 because of side effects and switched treatment arms, i.e., from MAOI to D2RAG (*n* = 2) or the reverse (*n* = 2), of whom two remitted (Figure 3, T2). If we include them, 32 (67%) patients stopped after DATA1: 22 Rm, 27 Rs (+5), and 5 nRs (Figure 3, T3). The five Rs/nRm who decided to stop after DATA1 were satisfied with the result and maintained the treatment. All were at the edge of Rm (QIDS = 6) and had a good functional outcome (GAF = 73 ± 6). Regarding nRs, one patient could neither take MAOI nor D2RAG at an adequate dose because of side effects (two or more drugs of each class had been tried). Of the four nRs who achieved adequate doses and duration, only one continued the treatment (QIDS = 10); the three others stopped and did not want to test another molecule because of poor efficacy relative to side effects (QIDS = 17 ± 4).

#### 3.2.3. DATA 2: combo

Only 16 patients proceeded to DATA2, of whom three were already Rm and three were Rs/nRm. All aimed at remission *ad integrum*. Accordingly, when entering DATA2, the average severity was mild to moderate (QIDS = 10 ± 4). Relative to the 32 patients who stopped after DATA1, patients who entered DATA2 tended to have more severe (*p* < 0.06) and more resistant depression at onset (*p* = 0.008, see Table 2, Section 2c).

At the end of DATA2, 15 patients were Rm and one patient remained nRs despite achieving adequate dose and duration. The average QIDS score = 3 ± 2 and the average GAF = 69 ± 15. MAOI were taken at average doses of 51 ± 16 mg/d TCP-eq, combined with D2RAG at 2.8 ± 2.3 mg/d PPX-eq. The time to remission was of 12 [9–25] weeks, which was significantly longer than in DATA1 (*p* = 0.02, Figure 3, T4).

#### 3.2.4. Deviations from DATA guidelines

Only 38 (79%) patients received a mood stabilizer during the period of interest. According to the DATA guidelines, 19 (40%) patients received lithium carbonate (discontinued in two patients during the DATA2 phase – see ‘*’, Table 4, Section 4c for details) and 12 (25%) were treated with lamotrigine. Some received potentially D2R blockers, even if at low doses: quetiapine (150 ± 100 mg/d; *n* = 4) and aripiprazole (10 mg/d; *n* = 2). One patient had clozapine (50 mg/d).

As already mentioned, because of side effects, four patients switched from MAOI to D2RAG or *vice-versa* rather than combining the drugs. As indicated in Figure 3, the two patients who switched from D2RAG to MAOI remitted while the two patients who switched from MAOI to D2RAG were nRs (including the patient for whom neither MAOI nor D2RAG could be maintained at a sufficient dose and duration). These patients were only mentioned for their first trial in Table 2 Section 2b but included in the global outcome (ITT and per-protocol analyses; Figure 3-T5, Table 4).

Finally, regarding the exclusion criteria, five (10%) patients were later diagnosed or suspected of having a slowly developing form of synucleinopathies (four definite Parkinson’s diseases, one probable prodromal form) (37). The single nRs after DATA2 subsequently developed cognitive and neurological disorders of probable mixed etiology (vascular and neurodegenerative), explaining the poor functional outcome despite mild symptom severity (Figure 3). Nine subjects had or developed significant personality traits with improvement, seven of whom fulfilled the ‘atypical’ criteria during the episode (see Supplementary material).

### 3.3. Intention-to-treat analysis

#### 3.3.1. Proportion of Rm, Rs, and nRs

Of the 48 patients with TRAD who entered DATA, 37 (77%) were Rm with a total time to remission of 25 [14–55] weeks, i.e., about 6 months (adding the time of DATA1 and DATA2 for Rm after the combination, Figure 3-T5). Though not formerly Rm according to the classical threshold, the five Rs who stopped after DATA1 while at the edge of remission (QIDS = 6) were closer to the group of Rm than the group of nRs: they felt their needs were met and had no or little functional impairment (GAF = 73 ± 6, i.e., similar to Rm = 74 ± 11 but significantly different from nRs = 44 ± 8; *p* = 9 × 10^−4^).

#### 3.3.2. Added value of DATA2

When comparing the treatments given separately (DATA1) to their combination (DATA1 + 2), the latter had a significant added value in terms of Rm (*n* = 25 → 37; *p* = 4 × 10^−5^), as well as in terms of Rs (*n* = 31 → 42; *p* = 2 × 10^−6^ – Table 3).

**Table 3:** Additional value of DATA2 (intention-to-treat analysis).

		DATA1	DATA2	*p*	OR
Total	Rm	52% [38–66%] (*n* = 25)	77% [65–89%] (*n* = 37)	**4 × 10**^**−5**^	**5.2**
(*n* = 48)	Rs	65% [51–78%] (*n* = 31)	88% [78–97%] (*n* = 42)	**2 × 10**^**−6**^	**5**
MAOI first	Rm	45% [29–61%] (*n* = 17)	76% [63–90%] (*n* = 29)	**5 × 10**^**−6**^	**6.8**
(*n* = 38)	Rs	66% [51–81%] (*n* = 25)	84% [73–96%] (*n* = 32)	**0.0018**	**3.9**
D2RAG first	Rm	60% [30–90%] (*n* = 6)	80% [55–100%] (*n* = 8)	*0.11*	**4.2**
(*n* = 10)	Rs				

*p*-values of *χ*^2^-tests. Rm, remitters; Rs, responders. The *p*-values are in bold when the difference is significant (*α* = 0.05, bilateral, uncorrected for multiple testing), and in gray italic when not significant. Odds ratios (OR) show that adding MAOI to D2RAG is similar in magnitude to adding D2RAG to MAOI.

When patients started with MAOI, the combination with a D2RAG resulted in a significant increase in Rm and Rs (*n* = 17 → 29 and *n* = 25 → 32; *p* = 5 × 10^−6^ and 2 × 10^−3^, respectively). Starting from D2RAG combined with a ‘classical’ antidepressant, the switch to a MAOI resulted in an increase of Rm (*n* = 6 → 8). This failed to reach significance likely due to the small sample size (*n* = 10) since the odds ratios are of the same magnitude (Table 3).

### 3.4. Duration of remission (survival analysis)

**Figure 4:**
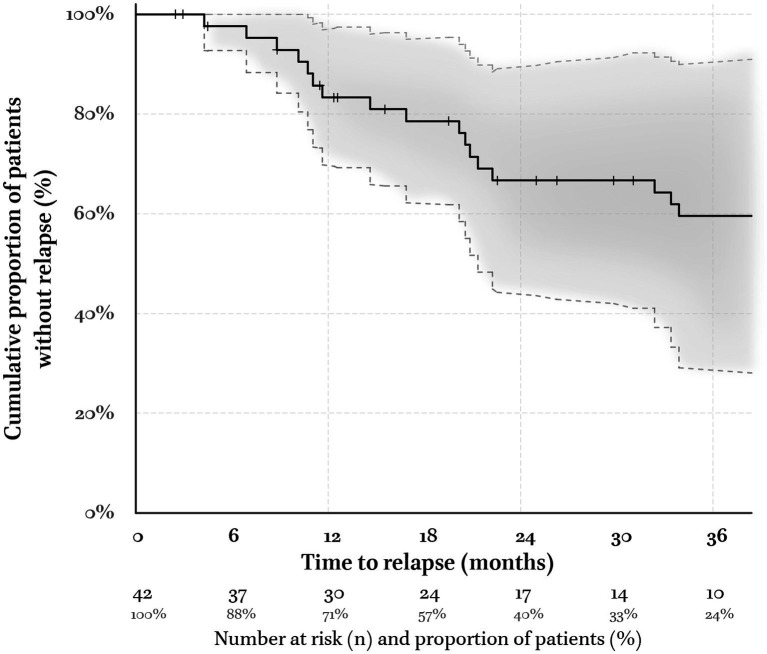
Kaplan–Meier estimator of the remission ‘survival’ function. The five responders who were not formally remitters according to their QIDS = 6 but who were satisfied with MAOI are included in the pool of remitters (Rm). Vertical tick-marks state patients who were still Rm at their last visit (right-censored survival). The numbers at risk and their proportion relative to the initial sample are indicated below the time abscise. The envelop corresponds to the 95% confidence interval (Gaussian approximation to the binomial distribution). At 6, 12, and 18 months, 95% [88–100%], 86% [73–98%], and 78% [61–95%] of the patients did not relapse, respectively. This compares best with cohorts treated in expert centers (see Supplementary material).

The 48 patients included in the analysis have been followed-up for a median of 3 [1.4–4.8] years since beginning DATA. The Kaplan–Meier estimator of the duration of remission considered 37 Rm and 5 Rs/nRm together. At 18 months, 79% [62–95%] of the sample remained in Rm (24 patients, i.e., 57% of the sample, remained in the analysis – Figure 4). Six out of nine relapses occurred when tapering antidepressant treatments (67%) and could be successfully treated by returning to therapeutic doses.

### 3.5. Predictors of Rm and Rs

If the resistance of depression predicted the outcomes of DATA1, this was no more the case for DATA2 and in the (global) ITT analysis. The only negative predictor of Rm or Rs in the ITT analysis was a concurrent diagnosis of DSM-atypical depression (Table 4, see Supplementary material for effect size and personality traits).

**Table 4:** Predictors of Rm and Rs with DATA.

Population characteristics	4.a – DATA1 (*n* = 48)			4.b – DATA2 (*n* = 16)		4.c – ITT (*n* = 48)					
	Rm	nRm	*p*	Rm	nRm	Rm	nRm	*p*	Rs	nRs	*p*
Population (*N*)	23 (48%)	25 (52%)		15 (94%)	1 (6%)	37 (77%)	11 (23%)		42 (88%)	6 (13%)	
Female/Male (% female)	10/13 (43%)	12/13 (48%)	–	8/7 (53%)	1/0	16/21 (43%)	6/5 (55%)	–	18/24 (43%)	4/2 (67%)	–
Age (years)	57 ± 13	60 ± 11	–	58 ± 12	84	57 ± 11	64 ± 13	–	59 ± 11	58 ± 13	–
Age at first episode (years)	41 ± 16	43 ± 16	–	43 ± 20	61	41 ± 16	48 ± 14	–	43 ± 17	38 ± 12	–
Number of previous episodes	2 ± 2	2 ± 2	–	3 ± 3	2	2 ± 2	1 ± 1	–	2 ± 2	1 ± 1	–
Bipolar disorder*	5 (22%)	9 (36%)	–	5 (33%)	1	10 (27%)	4 (36%)	–	11 (26%)	3 (50%)	–
Affected first degree relative	16 (70%)	14 (56%)	–	9 (60%)	0	25 (68%)	5 (45%)	–	27 (64%)	3 (50%)	–
Lead-in period (weeks)	16 (7–24)	20 (5–43)	–	9 (4–34)	45	14 (5–30)	22 (5–43)	–	13 (5–30)	32 (20–43)	–
Current episode resistance											
Duration of the episode (years)^¤^	4.5 ± 3.1	3.8 ± 2.4	–	3.7 ± 2.5	1.9	3.9 ± 2.7	4.5 ± 2.2	–	3.9 ± 2.7	5.8 ± 2.4	–
Thase and Rush resistance	2.6 ± 0.5	3.2 ± 0.9	**0.016**	3.5 ± 1	3	2.9 ± 0.7	3 ± 0.5	–	2.9 ± 0.7	2.8 ± 0.3	–
›SSRI	22 (96%)	24 (96%)	–	15 (100%)	0	36 (97%)	10 (91%)	–	41 (98%)	5 (83%)	–
›SNRI	19 (83%)	23 (92%)	–	15 (100%)	1	32 (86%)	10 (91%)	–	36 (86%)	6 (100%)	–
›Tricyclics antidepressants	13 (57%)	18 (72%)	–	9 (60%)	1	23 (62%)	8 (73%)	–	26 (62%)	5 (83%)	–
›Antipsychotic	10 (43%)	12 (48%)	–	10 (67%)	1	18 (49%)	4 (36%)	–	18 (43%)	4 (67%)	–
›Combination or augmentation	15 (65%)	22 (88%)	*0.061*	13 (87%)	1	27 (73%)	10 (91%)	–	31 (74%)	6 (100%)	–
›rTMS	0 (0%)	4 (16%)	**0.045**	2 (13%)	1	2 (5%)	2 (18%)	–	2 (5%)	2 (33%)	**0.018**
›ECT	0 (0%)	6 (24%)	**0.012**	5 (33%)	0	5 (14%)	1 (9%)	–	6 (14%)	0 (0%)	–
Clinical features											
QIDS-C baseline	16.2 ± 3.4	17.4 ± 3.1	–	16.2 ± 3.5	17	16.4 ± 3.4	16.6 ± 3.7	–	16.3 ± 3.3	17.5 ± 4.5	–
GAF baseline	40 ± 8	41 ± 8	–	38 ± 9	55	40 ± 9	45 ± 5	–	41 ± 8	42 ± 6	–
›Anxiety	17 (74%)	23 (92%)	*0.093*	13 (87%)	0	30 (81%)	10 (91%)	–	35 (83%)	5 (83%)	–
›Psychotic features	3 (13%)	5 (20%)	–	4 (27%)	0	8 (22%)	0 (0%)	*0.096*	8 (19%)	0 (0%)	–
›Mixed or incomplete states	19 (83%)	16 (64%)	–	11 (73%)	1	29 (78%)	6 (55%)	–	30 (71%)	5 (83%)	–
›Mood reactivity and/or fluctuations	20 (87%)	22 (88%)	–	12 (80%)	1	31 (84%)	11 (100%)	–	36 (86%)	6 (100%)	–
›Atypical criteria (DSM-5)	4 (17%)	9 (36%)	–	2 (13%)	0	6 (16%)	7 (64%)	**0.002**	9 (21%)	4 (67%)	**0.02**
›Increased appetite	5 (22%)	3 (12%)	–	3 (20%)	0	7 (19%)	1 (9%)	–	7 (17%)	1 (17%)	–
›Increased weight (≥10%)	5 (22%)	7 (28%)	–	5 (33%)	0	10 (27%)	2 (18%)	–	11 (26%)	1 (17%)	–
›Hypersomnia	8 (35%)	12 (48%)	–	9 (60%)	0	15 (41%)	5 (45%)	–	16 (38%)	4 (67%)	–
Treatment											
Number of patients taking Li + (%)	10 (42%)	7 (29%)	–	6 (40%)	1 (100%)	16/35 (46%)*	1 (9%)*	0.03*	16/40 (40%)*	1 (17%)*	–
MAOI (mg/d TCP-eq)	49 ± 19	49 ± 18	–	52 ± 16	50	52 ± 18	40 ± 18	–	51 ± 17	36 ± 23	–
D2RAG (mg/d PPX-eq)	1.9 ± 0	4.7 ± 2.2	*0.068*	3.2 ± 2.5	1	3.1 ± 2.3	1.8 ± 1.1	–	3 ± 2.3	2 ± 1.3	–
Results											
QIDS (end|final)	2.7 ± 1.8	11.3 ± 4	**8 × 10**^**−10**^	2.2 ± 1.4	8	2.5 ± 1.7	10.5 ± 4.6	**2 × 10**^**−8**^	2.9 ± 1.8	14.5 ± 4.7	**2 × 10**^**−11**^
Time to Rm|trial completion (weeks)	11 (7–15)	4 (3–26)	–	12 (4–25)	–	14 (6–93)	142 (27–294)	**0.008**	16 (6–93)	153 (138–294)	**0.009**
GAF final	75 ± 10	–	–	72 ± 13	30	74 ± 11	57 ± 16	**0.001**	74 ± 10	44 ± 8	**2 × 10**^**−6**^

### 3.6. Tolerance

After drug and dose optimization in DATA1, 30 patients presented at least one side effect (63% [49–76%]), 26 patients under MAOI and four under D2RAG (72% [58–87%] vs. 40% [10–70%]; *p* = 0.058). This trend vanishes when the four switchers are included: 26 under MAOI and six under D2RAG (68% [54–83%] vs. 50% [22–78%]). In DATA2, after drug and dose optimization, 13 patients presented at least one side effect (81% [62–100%]). Drug and dose adaptation strategies have been detailed in a previous paper (27). None of these side effects were serious adverse reactions.

## 4. Discussion

The main findings of our study are the good remission and response rates in patients with TRAD by using dopaminergic treatments as the first line, and the significant added value of their combination and their maintenance in time. We will first compare these results to the literature before discussing their limits.

### 4.1. DATA1 comparison with the literature

**Figure 5:**
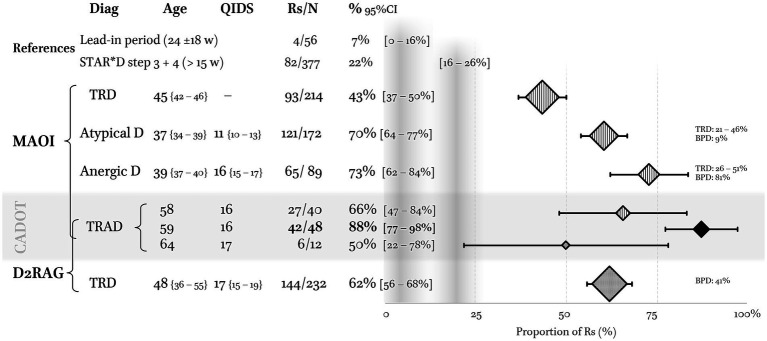
Comparison of the proportion of responders (Rs) in CADOT with the literature. The first reference (gray vertical band) is our lead-in period of 24 weeks during which four patients remitted with other treatment than DATA (but three remitted with bupropion + SSRI, i.e., a dopaminergic strategy). The second is the proportion of Rs when combining stages 3 and 4 of the STAR*D trial (38–40). Regarding monoamine oxidase inhibitors (MAOI), the results in non-stratified treatment-resistant depression (TRD) were extracted from (41). MAOI in atypical depression: weighted average of Rs from an intention-to-treat perspective (see references in text). Proportion of TRD and bipolar depressions (BPD) are indicated on the right. MAOI in anergic depression: weighted average of Rs from an intention-to-treat perspective. All studies are from the Pittsburgh group (see references in text). The single dopamine D2 receptor agonist (D2RAG) on which we have sufficient data is pramipexol (PPX). The proportion of Rs with PPX in TRD is recomputed from 10 studies reviewed in reference (42).

In the absence of a control group, absolute values of Rs (and Rm) may be interpreted relative to two references: (i) the number of Rm with other attempts implemented during the nearly half-year lead-in period (23 ± 18 w), i.e., 7% [0–14%] (Figure 2), and (ii) the proportion of Rs when combining stages 3 and 4 of the STAR*D trial, i.e., 22% [18–26%] over an average of 15 weeks (Figure 5, shaded areas). These included a switch to other antidepressants (nortriptyline, mirtazapine, or TCP), augmentation (lithium or triiodothyronine) and combination strategies (venlafaxine + mirtazapine) (38–40).

In the following section, comparisons are performed on fixed-effect meta-analyses of Rs reported in the literature (ITT analysis, i.e., including dropouts, Figure 5). Only randomized controlled studies are considered when discussing MAOI but not when considering D2RAG.

#### 4.1.1. Switching to a MAOI

In non-stratified TRD, our results are better than expected when compared with the seven studies using TCP: 68% [50–85%] vs. 43% [37–50%]. This difference can neither be accounted for by differences in severity, resistance, doses, or treatment duration, all being in the same range (41). Hence, there might be an advantage to stratify TRD patients. In the following, TRAD will be compared with two other clinical subgroups: atypical depression and anergic depression.

In the seven studies on atypical depression, the weighted average of Rs to phenelzine was 61% [54–67%] (18, 43–48). Two thirds of these patients were chronically depressed, and many may have suffered from dysthymia or double depression regarding the moderate level of severity (QIDS ≈ 11) and resistance (22–48% of TRD). Hence, our results stand up well since the sample had much less favorable characteristics and only half of our patients had atypical features. In CADOT, 83% of the patients received TCP rather than phenelzine and received higher doses, i.e., 50 mg/d vs. 40 mg/d of TCP-eq (59 mg/d of phenelzine). This difference in doses has probably less to do with the nature of the drug than with the naturalistic setting allowing to take the time needed for dose optimization and to maximize tolerance. In CADOT, only 5% of the patients could not achieve the appropriate dose and duration vs. 19% in the randomized control trials on atypical depression. If the proportion of Rs is computed on the number of patients who received an adequate treatment rather than the number of patients at inclusion, i.e., per-protocol rather than ITT analysis, there is no more difference: 71% [65–78%] in atypical depression vs. 69% in our study.

All the four studies in anergic depression are from the Pittsburgh group in which the weighted average of Rs to TCP was 73% [62–84%] (14–16, 19). They mostly recruited inpatients who were more often bipolar (49% vs. 35%), but they had similar symptom severity and were less resistant (<51% vs. 100%). Again, the rigid timeframe of the Pittsburgh’s randomized controlled trials accounted for the lower doses and shorter treatment duration before evaluation relative to CADOT (35 vs. 50 mg/d TCP-eq; 6 vs. 15 weeks).

#### 4.1.2. PPX (D2RAG) in TRD

Of the ten reports on the use of PPX in TRD, three were randomized control trials, three included bipolar patients, and only one addressed its use in combination with a (non-MAOI) antidepressant as in DATA1 (42). None questioned the added value of D2RAG in specific TRD sub-types. Our results of PPX in DATA1 are below the range of those in the published studies, i.e., 50% [22–78%] vs. 62% [56–68%] despite the use of (much) higher doses, 3.6 mg/d vs. 1.4 mg/d (26).

#### 4.1.3. Interim conclusion

The average proportion of Rs of 65% after DATA1 supports the added value of considering TRAD as most likely to benefit from a dopaminergic antidepressant strategy.

### 4.2. The benefit of DATA2 combination

To the best of our knowledge, this is the first report of the combination of MAOI and D2RAG in the management of TRD (notwithstanding the anergic-anhedonic features) (49). When considering only the group of patients who entered DATA2, the proportion of Rm was unexpectedly high both in the whole sample (*n* = 16) and when considered in only the subgroup of nRm (*n* = 13), i.e., 94% [82–100%] and 92% [78–100%], respectively, being fully remitted (QIDS = 2, Table 4, Section 4b). In line with earlier reports on a similar clinical picture (13), it is of interest that all the five ECT-resistant patients who entered DATA2 remitted, suggesting that this might not be of unfavorable prognosis in the case of anergic-anhedonic depression.

However, the results of DATA2 cannot be interpreted on their own. The significant proportion of nRm after DATA1 who refused to enter DATA2 benefited less from the first line of treatment (QIDS = 16) than those who continued (QIDS = 10 – Figure 3). A complete absence of response to a first dopaminergic agent may have been interpreted by the patient and his/her psychiatrist as predictive of a low probability of response to the continuation of this strategy. Lastly, lithium discontinuation in two patients may have contributed to the improvement (‘*’ Figure 4C). Accordingly, DATA efficacy on TRAD can only be assessed from an ITT perspective. Though somewhat less striking, the increase of Rm from 52 to 77% and of Rs from 65 to 88% remains significant.

### 4.3. Efficiency of treatment maintenance

Even if all patients with a QIDS score ≤6 could not be maintained under the same treatment and the same doses up to 6 months, 95% [88–100%] of them did not relapse. This is much higher than the 6 months follow-up after steps 3 and 4 of the STAR*D trial in which only 38% [22–55%] were still in remission (4). Conversely, our maintenance rate is consistent with the 80% [71–90%] (*n* = 71/57) of TRD patients followed up in tertiary care (50) (detailed in the Supplementary material). Interestingly, these authors also reported a positive association between maintenance with MAOI and the absence of relapse.

### 4.4. Limitations

#### 4.4.1. TRAD in the context of French psychiatric care

It is uneasy to ascertain the generalizability of these results out of the context of French psychiatry. In our region, ECT was only available in an inpatient setting and the specificities of French health insurance coverage might have biased our population with TRAD.

In France, bupropion, the only norepinephrine-dopamine reuptake inhibitor available, is not reimbursed for depression. Yet its dopaminergic valence could make it particularly beneficial in cases of anergic-anhedonic depression (51). As bupropion is hardly ever used for depression in France, it may be that TRAD are not only more frequent, but also less resistant to dopaminergic antidepressant strategies relative to other countries in which bupropion is a second line treatment, alone or in combination (52–54). In CADOT, only five patients accepted to support the cost of bupropion and tried it in combination with the SNRI duloxetine before trying DATA; three of them remitted with the association, so that only two entered DATA – of whom all were Rm.

Furthermore, since structured psychotherapy is not reimbursed too, only two of our patients had an adequate trial of cognitive-behavioral therapy before trying DATA. This may have been particularly beneficial to patients with atypical features (48) and may account for the poor prognostic value of this characteristic in our ITT analysis (see Table 4, Section 4c, and the Supplementary material for personality traits). Moreover, the most effective psychotherapy for chronic depression, CBASP (Cognitive Behavioral Analysis System of Psychotherapy), is not available in France (55).

#### 4.4.2. Limitations related to the prospective observational design

Regarding DATA1, it is impossible to conclude a superiority of MAOI or D2RAG as the first line treatment (as it was non-significant whether switchers were or were not included). Samples were not randomized because the decision was up to the patient, although the psychiatrist might have also played a role. For example, the difference in resistance between the MAOI and D2RAG groups (Table 4, Section 4a) likely reflects the prescriber’s belief in the higher chance of success with the former.

Overall, as enthusiastic as they may seem, the results of DATA1-2 benefited from the high motivation of patients and their caregivers and the flexibility of the prescribing context. Considering that the treatment would have to be maintained after remission, prescribers were particularly careful to optimize tolerance from the very start. Depending on the side effects and the patient’s preference, either the dose escalation was adapted or the molecule was switched to another from the same class. Only quaternary care facilities permit practitioners to devote the necessary time to do so, which may account for the low proportion of patients who could not have at least one adequate trial (*n* = 1, 2% [0–6%]).

Moreover, the results may have been favorably influenced by the flexible evaluation schedule and the long lead-in period (a median of 4 months). On the one hand, this allowed the establishment of the relationship of trust necessary for patients to accept riskier drugs and ascertained the resistance of the depression (when other lines of treatment were tried). On the other hand, it may have increased expectations and the placebo effect, not to mention that our belief in DATA for TRAD may sometimes have led us to convince the patient to pursue the trial where neutral prescribers would not have insisted.

Finally, the analysis of the whole TRAD cohort (see Supplementary material for an example) suggested some improvements to the description and the model of mesencephalic hypodopaminergia (Foucher et al., in preparation). The refinement of this dopamine-sensitive anergic-anhedonic syndrome could benefit from brain imaging biomarkers which would facilitate the implementation of multicenter randomized control trials. Without them, DATA-like guidelines are not recommended before traditional resources are exhausted and out of the hands of trained prescribers.

## 5. Conclusion

These results are not the first to suggest the existence of an anergic-anhedonic depressive syndrome distinct by its sensitivity to MAOI. By extending this sensitivity to D2RAG, they lend support to the hypothesis of mesencephalic hypodopaminergia as pathophysiological substratum. It is all the more timely to establish the existence of such an anergic-anhedonic syndrome and its relationship with ‘positive valence systems’, as new pro-dopaminergic interventions are emerging, e.g., triple reuptake inhibitors (56) and deep brain stimulation of the medial forebrain bundle (57).

## Data availability statement

The raw data supporting the conclusions of this article will be made available by the authors, without undue reservation.

## Ethics statement

The studies involving humans were approved by comite d’ethique des Facultés de Médecine, d’Odontologie, de Pharmacie, des Ecoles d’Infirmières, de Kinésithérapie, de Maïeutique et des Hôpitaux de Strasbourg – reference no. CE-2023-31. The studies were conducted in accordance with the local legislation and institutional requirements. Written informed consent for participation was not required from the participants or the participants’ legal guardians/next of kin because All patients were directly contacted and consented to the use of their data.

## Author contributions

JF and GB contributed to the conception and design of the study. LD-J organized the database. JF and LD-J performed the statistical analysis and wrote the first draft of the manuscript. JF and OM collected the data. All authors contributed to the article and approved the submitted version.

## Conflict of interest

The authors declare that the research was conducted in the absence of any commercial or financial relationships that could be construed as a potential conflict of interest.

## Publisher’s note

All claims expressed in this article are solely those of the authors and do not necessarily represent those of their affiliated organizations, or those of the publisher, the editors and the reviewers. Any product that may be evaluated in this article, or claim that may be made by its manufacturer, is not guaranteed or endorsed by the publisher.

## Supplementary material

The Supplementary material for this article can be found online at: https://www.frontiersin.org/articles/10.3389/fpsyt.2023.1194090/full#supplementary-material

## Figures and Tables

**Table:** 

CADOT	Chronic Anergic-anhedonic Depression Open Trial
D2RAG	Dopamine D2 receptor agonists
DATA	Dopaminergic Antidepressant Therapy Algorithm
GAF	Global Assessment of Functioning Scale
ITT	Intention-to-treat (analysis)
MAOI	Monoamine oxidase inhibitor (i.e., non-selective and irreversible)
PPX(–eq)	Pramipexole (equivalent doses)
QIDS	Quick Inventory of Depressive Symptomatology (clinician rated version, 16 items)
Rm/nRm	Remitter/non-remitter
Rs/nRs	Responder/non-responder (Rm or ≥50% QIDS reduction)
SNRI	Serotonin and norepinephrine reuptake inhibitor
TCP(–eq)	Tranylcypromine (equivalent doses)
TRAD	Treatment-resistant anergic-anhedonic depression
TRD	Treatment-resistant depression

## References

[1] Kennedy SH Lam RW McIntyre RS Tourjman SV Bhat V Blier P. Canadian network for mood and anxiety treatments (CANMAT) 2016 clinical guidelines for the management of adults with major depressive disorder: section 3 pharmacological treatments. Can J Psychiatry. (2016) 61:540–60. doi: 10.1177/0706743716659417, PMID: 27486148 PMC4994790

[2] NICE NI for H and CE. Depression in adults: recognition and management. Clin Guidel [NG222] (2022), Available at: https://www.nice.org.uk/guidance/ng222

[3] Ruberto VL Jha MK Murrough JW. Pharmacological treatments for patients with treatment-resistant depression. Pharmaceuticals. (2020) 13:116. doi: 10.3390/ph13060116, PMID: 32512768 PMC7345023

[4] Rush AJ Trivedi MH Wisniewski SR Nierenberg AA Stewart JW Warden D. Acute and longer-term outcomes in depressed outpatients requiring one or several treatment steps: a STAR*D report. Am J Psychiatry. (2006) 163:1905–17. doi: 10.1176/ajp.2006.163.11.1905, PMID: 17074942

[5] Pigott HE. The STAR*D trial: it is time to reexamine the clinical beliefs that guide the treatment of major depression. Can J Psychiatr. (2015) 60:9–13. doi: 10.1177/070674371506000104, PMID: 25886544 PMC4314062

[6] Kupfer DJ First MB Regier DA. Introduction, A research agenda for DSM-V, (Eds.) Kupfer D. J. First M. B. Regier D. A. (Washington, D.C.: American Psychiatric Association), xviii–xix. Available at: https://www.appi.org/Research_Agenda_For_DSM_V (Accessed May 12, 2017)

[7] Foucher J-R Bennouna GV. ICD and DSM, the invalidable. Ann Med Psychol (Paris). (2010) 168:609–15. doi: 10.1016/j.amp.2009.12.018

[8] Lynch CJ Gunning FM Liston C. Causes and consequences of diagnostic heterogeneity in depression: paths to discovering novel biological depression subtypes. Biol Psychiatry. (2020) 88:83–94. doi: 10.1016/j.biopsych.2020.01.012, PMID: 32171465

[9] Wium-Andersen IK Vinberg M Kessing LV McIntyre RS. Personalized medicine in psychiatry. Nord J Psychiatry. (2017) 71:12–9. doi: 10.1080/08039488.2016.1216163 27564242

[10] Chaudhuri KR Schapira AHV. Non-motor symptoms of Parkinson’s disease: dopaminergic pathophysiology and treatment. Lancet Neurol. (2009) 8:464–74. doi: 10.1016/S1474-4422(09)70068-7, PMID: 19375664

[11] Thobois S Lhommée E Klinger H Ardouin C Schmitt E Bichon A. Parkinsonian apathy responds to dopaminergic stimulation of D2/D3 receptors with piribedil. Brain. (2013) 136:1568–77. doi: 10.1093/brain/awt067, PMID: 23543483

[12] Thobois S Ardouin C Lhommée E Klinger H Lagrange C Xie J. Non-motor dopamine withdrawal syndrome after surgery for Parkinson’s disease: predictors and underlying mesolimbic denervation. Brain. (2010) 133:1111–27. doi: 10.1093/brain/awq032, PMID: 20237128

[13] Alexander L Berkeley AW. The inert psychasthenic reaction (anhedonia) as differentiated from classic depression and its response to iproniazid. Ann N Y Acad Sci. (1959) 80:669–79. doi: 10.1111/j.1749-6632.1959.tb49244.x, PMID: 13792590

[14] Himmelhoch JM Detee T Kupfer DJ Swartzburg M Byck R. Treatment of previously intractable depressions with tranylcypromine and lithium. J Nerv Ment Dis. (1972) 155:216–20. doi: 10.1097/00005053-197209000-00009, PMID: 5053920

[15] Himmelhoch J Thase M Mallinger A Houck P. Tranylcypromine versus imipramine in anergic bipolar depression. Am J Psychiatry. (1991) 148:910–6. doi: 10.1176/ajp.148.7.910, PMID: 2053632

[16] Thase ME Mallinger AG McKnight D Himmelhoch JM. Treatment of imipramine-resistant recurrent depression, IV: a double-blind crossover study of tranylcypromine for anergic bipolar depression. Am J Psychiatry. (1992) 149:195–8. doi: 10.1176/ajp.149.2.195, PMID: 1734739

[17] Liebowitz MR Quitkin FM Stewart JW McGrath PJ Harrison W Rabkin J. Phenelzine vs imipramine in atypical depression: a preliminary report. Arch Gen Psychiatry. (1984) 41:669–77. doi: 10.1001/archpsyc.1984.01790180039005, PMID: 6375621

[18] Liebowitz MR Quitkin FM Stewart JW McGrath PJ Harrison WM Markowitz JS. Antidepressant specificity in atypical depression. Arch Gen Psychiatry. (1988) 45:129–37. doi: 10.1001/archpsyc.1988.01800260037004, PMID: 3276282

[19] Himmelhoch JM Fuchs CZ Symons BJ. A double-blind study of tranylcypromine treatment of major anergic depression. J Nerv Ment Dis. (1982) 170:628–34. doi: 10.1097/00005053-198210000-00007, PMID: 7050302

[20] Papakostas GI. Dopaminergic-based pharmacotherapies for depression. Eur Neuropsychopharmacol. (2006) 16:391–402. doi: 10.1016/j.euroneuro.2005.12.002, PMID: 16413172

[21] Leonhard K. Etiology of endogenous psychoses, Classification of endogenous psychoses and their differentiated etiology (Wien, New-York: Springer), 279–329

[22] Foucher J Gawlik M Roth JN de Billy C Jeanjean LC Obrecht A. Wernicke-Kleist-Leonhard phenotypes of endogenous psychoses: a review of their validity. Dialogues Clin Neurosci. (2020) 22:37–49. doi: 10.31887/DCNS.2020.22.1/jfoucher, PMID: 32699504 PMC7365293

[23] Ghaemi SN Saggese J Goodwin FK. Diagnosis of bipolar depression, Bipolar depression: a comprehensive guide, (Eds.) El-Mallakh R. S. Ghaemi S. N. (Washington, DC: American Psychiatric Pub), 3–36

[24] Ghaemi SN. Why antidepressants are not antidepressants: STEP-BD, STAR*D, and the return of neurotic depression. Bipolar Disord. (2008) 10:957–68. doi: 10.1111/j.1399-5618.2008.00639.x, PMID: 19594510

[25] MacKenzie EM Song M-S Dursun SM Tomlinson S Todd KG Baker GB. Phenelzine: an old drug that may hold clues to the development of new neuroprotective agents. Klin Psikofarmakol Bülteni-Bulletin Clin Psychopharmacol. (2010) 20:179–86. doi: 10.1080/10177833.2010.11790656

[26] Fawcett J Rush AJ Vukelich J Diaz SH Dunklee L Romo P. Clinical experience with high-dosage pramipexole in patients with treatment-resistant depressive episodes in unipolar and bipolar depression. Am J Psychiatry. (2016) 173:107–11. doi: 10.1176/appi.ajp.2015.15060788, PMID: 26844792

[27] Dormegny-Jeanjean L Mainberger O de Billy C Obrecht A Danila V Erb A. Safety and tolerance of MAOI and dopamine agonists combination in depression. Encéphale. (2023). doi: 10.1016/j.encep.2023.01.011, PMID: 37005193

[28] Thase M Rush A. When at first you don’t succeed: sequential strategies for antidepressant nonresponders. J Clin Psychiatry. (1997) 58:23–9. PMID: Available at: https://www.psychiatrist.com/jcp/depression/dont-succeed-sequential-strategies-antidepressant/9402916

[29] Rush AJ Trivedi MH Ibrahim HM Carmody TJ Arnow B Klein DN. The 16-item quick inventory of depressive symptomatology (QIDS), clinician rating (QIDS-C), and self-report (QIDS-SR): a psychometric evaluation in patients with chronic major depression. Biol Psychiatry. (2003) 54:573–83. doi: 10.1016/S0006-3223(02)01866-8, PMID: 12946886

[30] Hall RC. Global assessment of functioning – a modified scale. Psychosomatics. (1995) 36:267–75. doi: 10.1016/S0033-3182(95)71666-8, PMID: 7638314

[31] Fiedorowicz JG Swartz KL. The role of monoamine oxidase inhibitors in current psychiatric practice. J Psychiatr Pract. (2004) 10:239–48. doi: 10.1097/00131746-200407000-00005, PMID: 15552546 PMC2075358

[32] Pare CMB Sandler M. A clinical and biochemical study of a trial of iproniazid in the treatment of depression. J Neurol Neurosurg Psychiatr. (1959) 22:247 LP–251. doi: 10.1136/jnnp.22.3.247 PMC497384 14430381

[33] Thobois S. Proposed dose equivalence for rapid switch between dopamine receptor agonists in Parkinson’s disease: a review of the literature. Clin Ther. (2006) 28:1–12. doi: 10.1016/j.clinthera.2005.12.003, PMID: 16490575

[34] Chung SJ Asgharnejad M Bauer L Benitez A Boroojerdi B Heidbrede T. Switching from an oral dopamine receptor agonist to rotigotine transdermal patch: a review of clinical data with a focus on patient perspective. Expert Rev Neurother. (2017) 17:737–49. doi: 10.1080/14737175.2017.1336087, PMID: 28548894

[35] Foucher J Robert A de Billy C Obrecht A Weibel S Bertschy G. Personalized rTMS reduce functional brain anomalies in resistant depression while classical rTMS and tDCS have no effect: preliminary results. Brain Stimul. (2019) 12:580–1. doi: 10.1016/j.brs.2018.12.926

[36] American Psychiatric Association. DSM 5. Washington DC, USA: American Psychiatric Publishing (2013) doi: 10.1176/appi.books.9780890425596.744053

[37] Jeanjean LC Mainberger O de Billy C Obrecht A Landré L Weibel S. Dépression anergique: différence entre phénotypes bipolaire et synucléinopathique? French J Psychiatry. (2019) 1:S109. doi: 10.1016/j.fjpsy.2019.10.335

[38] Fava M Rush AJ Wisniewski SR Nierenberg AA Alpert JE McGrath PJ. A comparison of mirtazapine and nortriptyline following two consecutive failed medication treatments for depressed outpatients: a STAR*D report. Am J Psychiatry. (2006) 163:1161–72. doi: 10.1176/ajp.2006.163.7.1161, PMID: 16816220

[39] Nierenberg AA Fava M Trivedi MH Wisniewski SR Thase ME McGrath PJ. A comparison of lithium and T(3) augmentation following two failed medication treatments for depression: a STAR*D report. Am J Psychiatry. (2006) 163:1519–30. doi: 10.1176/ajp.2006.163.9.1519, PMID: 16946176

[40] McGrath PJ Stewart JW Fava M Trivedi MH Wisniewski SR Nierenberg AA. Tranylcypromine versus venlafaxine plus mirtazapine following three failed antidepressant medication trials for depression: a STAR*D report. Am J Psychiatry. (2006) 163:1531–41. doi: 10.1176/ajp.2006.163.9.1531, PMID: 16946177

[41] Ricken R Ulrich S Schlattmann P Adli M. Tranylcypromine in mind (part II): review of clinical pharmacology and meta-analysis of controlled studies in depression. Eur Neuropsychopharmacol. (2017) 27:714–31. doi: 10.1016/j.euroneuro.2017.04.003, PMID: 28579071

[42] Tundo A de Filippis R De Crescenzo F. Pramipexole in the treatment of unipolar and bipolar depression. A systematic review and meta-analysis. Acta Psychiatr Scand. (2019) 140:116–25. doi: 10.1111/acps.13055, PMID: 31111467

[43] Quitkin FM Stewart JW McGrath PJ Liebowitz MR Harrison WM Tricamo E. Phenelzine versus imipramine in the treatment of probable atypical depression: defining syndrome boundaries of selective MAOI responders. Am J Psychiatry. (1988) 145:306–11. doi: 10.1176/ajp.145.3.306, PMID: 3278631

[44] Quitkin F McGrath P Stewart J Harrison W Wager S Nunes E. Phenelzine and imipramine in mood reactive depressives: further delineation of the syndrome of atypical depression. Arch Gen Psychiatry. (1989) 46:787–93. doi: 10.1001/archpsyc.1989.01810090029005, PMID: 2673130

[45] Quitkin FM McGrath PJ Stewart JW Harrison W Tricamo E Wager SG. Atypical depression, panic attacks, and response to imipramine and phenelzine: a replication. Arch Gen Psychiatry. (1990) 47:935–41. doi: 10.1001/archpsyc.1990.01810220051006, PMID: 2222132

[46] Quitkin FM Harrison W Stewart JW McGrath PJ Tricamo E Ocepek-Welikson K. Response to phenelzine and imipramine in placebo nonresponders with atypical depression. A new application of the crossover design. Arch Gen Psychiatry. (1991) 48:319–23. doi: 10.1001/archpsyc.1991.01810280035005, PMID: 2009033

[47] Pande AC Birkett M Fechner-Bates S Haskett RF Greden JF. Fluoxetine versus phenelzine in atypical depression. Biol Psychiatry. (1996) 40:1017–20. doi: 10.1016/0006-3223(95)00628-1, PMID: 8915561

[48] Jarrett RB Schaffer M McIntire D Witt-Browder A Kraft D Risser RC. Treatment of atypical depression with cognitive therapy or phenelzine: a double-blind, placebo-controlled trial. Arch Gen Psychiatry. (1999) 56:431–7. doi: 10.1001/archpsyc.56.5.431, PMID: 10232298 PMC1475805

[49] Thomas SJ Shin M McInnis MG Bostwick JR. Combination therapy with monoamine oxidase inhibitors and other antidepressants or stimulants: strategies for the management of treatment-resistant depression. Pharmacother J Hum Pharmacol Drug Ther. (2015) 35:433–49. doi: 10.1002/phar.1576, PMID: 25884531

[50] Fekadu A Rane LJ Wooderson SC Markopoulou K Poon L Cleare AJ. Prediction of longer-term outcome of treatment-resistant depression in tertiary care. Br J Psychiatry. (2012) 201:369–75. doi: 10.1192/bjp.bp.111.102665, PMID: 22955008

[51] Walsh AEL Huneke NTM Brown R Browning M Cowen P Harmer CJ. A dissociation of the acute effects of bupropion on positive emotional processing and reward processing in healthy volunteers. Front. Psychiatry. (2018) 9:482. doi: 10.3389/fpsyt.2018.00482, PMID: 30386259 PMC6198095

[52] Lam RW Hossie H Solomons K Yatham LN. Citalopram and bupropion-SR: combining versus switching in patients with treatment-resistant depression. J Clin Psychiatry. (2004) 65:337–40. doi: 10.4088/JCP.v65n0308, PMID: 15096072

[53] Trivedi MH Fava M Wisniewski SR Thase ME Quitkin F Warden D. Medication augmentation after the failure of SSRIs for depression. N Engl J Med. (2006) 354:1243–52. doi: 10.1056/NEJMoa052964, PMID: 16554526

[54] Mohamed S Johnson GR Chen P Hicks PB Davis LL Yoon J. Effect of antidepressant switching vs augmentation on remission among patients with major depressive disorder unresponsive to antidepressant treatment: the VAST-D randomized clinical trial. JAMA. (2017) 318:132–45. doi: 10.1001/jama.2017.8036, PMID: 28697253 PMC5817471

[55] Kramer U Belz M Caspar F. Psychothérapie de la dépression chronique: l’apport du modèle CBASP selon McCullough. Encéphale. (2013) 39:137–42. doi: 10.1016/j.encep.2012.03.006, PMID: 23107463

[56] Mi W Yang F Li H Xu X Li L Tan Q. Efficacy, safety, and tolerability of ansofaxine (LY03005) extended-release tablet for major depressive disorder: a randomized, double-blind, placebo-controlled, dose-finding, phase 2 clinical trial. Int J Neuropsychopharmacol. (2022) 25:252–60. doi: 10.1093/ijnp/pyab074, PMID: 34747448 PMC8929756

[57] Coenen VA Bewernick BH Kayser S Kilian H Boström J Greschus S. Superolateral medial forebrain bundle deep brain stimulation in major depression: a gateway trial. Neuropsychopharmacology. (2019) 44:1224–32. doi: 10.1038/s41386-019-0369-9, PMID: 30867553 PMC6785007

