# Supplementary material for: Potential efficacy of dopaminergic antidepressants in treatment resistant anergic-anhedonic depression results of the chronic anergic-anhedonic depression open trial – CADOT
Source: Front Psychiatry. 2023 Sep 27;14:1194090. doi: 10.3389/fpsyt.2023.1194090 (PMC10565009; doi:10.3389/fpsyt.2023.1194090)
Supplement: Supplementary file 1 [file Data_Sheet_1.PDF]

Supplementary materials

Potential efficacy of dopaminergic antidepressants in treatment resistant anergic-anhedonic depression

Results of the Chronic Anergic-anhedonic Depression Open Trial – CADOT

Dormegny-Jeanjean LC, de Billy C, Mainberger OAE, Weibel S, Schorr B, Obrecht A, Landré L, Berna F, Causin J-B, Blanc F, Danila V, Tomsa M, Pfleger G, Meyer C, Humbert I, Javelot H, Meyer G, Bertschy G, Foucher JR*

Content

Effect size 1
Method for computing effect size 1
Table 2 – effect size 2
Table 4 – Effect size 4
Time to relapse – comparison between studies 6
Methods 6
Results 6
Population characteristics 6
Survival curves 7
Personality traits in CADOT 8
Methods 8
Results 8
At inclusion 8
Residual personality during the follow-up 8
Remark 9
Bibliography 10

* **Corresponding author:** Jack R Foucher. CEMNIS (UF 4768) - CEntre de neuroModulation Non-Invasive de Strasbourg. jack.foucher@unistra.fr. 1 place de l'Hôpital - BP 426, 67091 STRASBOURG Cedex, France, Tel: + 33 3 88 11 69 21

Effect size

Method for computing effect size

To homogenize the presentation of the magnitude of effect, we used the same metric, i.e. *d*.

For continuous data we computed the classical Cohen's *d* (implicitly assuming Gaussian distributions), i.e. 'normalized' distance between the means of the two groups $(\overline{x_1} - \overline{x_2})$(1):

$$d = \frac{\overline{x_1} - \overline{x_2}}{s_p}$$

With s_p the pooled standard deviation:

$$s_p = \sqrt[2]{\frac{(n_1 - 1) \cdot s_1^2 + (n_2 - 1) \cdot s_2^2}{n_1 + n_2 - 1}}$$

For categorial data we used the transformation of the OR (odd ratio) proposed by Hasselblad and Hedges (implicitly assuming logistic distributions and homogeneous variances)(2):

$$d_{HH} = \log(OR) \cdot \frac{\sqrt[2]{3}}{\pi}$$

Table 2 – effect size

	2.a. General	2.b. DATA1			2.c. DATA2		
**Population characteristics**		MAOI	D2RAG	*d*	No	Yes	*d*
Population (N)	48	38 (79%)	10 (21%)		32 (67%)	16 (33%)	
Femal / Male (% female)	22/26 (46%)	21/17 (55%)	1/9 (10%)	0.58	13/19 (41%)	9/7 (56%)	0.15
Age (years)	59 ±12	58 ±13	63 ±8	0.10	58 ±11	60 ±13	0.48
Age at first episode (years)	42 ±16	41 ±17	45 ±17	0.28	40 ±15	44 ±20	0.20
Number of previous episodes	3 ±3	3 ±3	2 ±2	0.32	3 ±3	3 ±3	0.04
Bipolar disorder*	14 (29%)	12 (32%)	2 (20%)	0.15	8 (25%)	6 (38%)	0.14
Affected first degree relative	30 (63%)	22 (58%)	8 (80%)	0.26	21 (66%)	9 (56%)	0.09
Lead-in period (weeks)	19 (5 – 37)	15 (2 – 37)	8 (2 – 23)	0.16	19 (3 – 30)	6 (1 – 34)	0.20
**Resistance of current episode**							
Duration of index episode (years)¤	4.1 ±2.7	4.1 ±2.5	4.2 ±3.8	0.02	4.4 ±2.8	3.6 ±2.4	0.29
Thase and Rush resistance	2.9 ±0.6	3.1 ±0.7	2.2 ±0.7	1.35	2.6 ±0.6	3.5 ±1	1.16
› SSRI	46 (96%)	37 (97%)	9 (90%)	0.34	31 (97%)	15 (94%)	0.17
› SNRI	42 (88%)	34 (89%)	8 (80%)	0.18	26 (81%)	16 (100%)	–
› Tricyclics antidepressants	31 (65%)	25 (66%)	6 (60%)	0.06	21 (66%)	10 (63%)	0.03
› Antipsychotic	22 (46%)	19 (50%)	3 (30%)	0.20	11 (34%)	11 (69%)	0.34
› Combination or augmentation	37 (77%)	31 (82%)	6 (60%)	0.26	23 (72%)	14 (88%)	0.24
› rTMS	4 (8%)	3 (8%)	1 (10%)	0.06	1 (3%)	3 (19%)	0.47
› ECT	6 (13%)	6 (16%)	0 (0%)	–	1 (3%)	5 (31%)	0.63
**Clinical features**							
QIDS-C baseline	16.4 ±3.4	16.2 ±3.4	17.1 ±3.2	0.29	15.4 ±3.2	17.8 ±3.3	0.73
GAF baseline	41 ±8	40 ±8	42 ±9	0.21	42 ±8	39 ±9	0.30
› Anxiety	40 (83%)	31 (82%)	9 (90%)	0.17	27 (84%)	13 (81%)	0.05
› Psychotic features	8 (17%)	4 (11%)	4 (40%)	0.42	4 (13%)	4 (25%)	0.20
› Mixed or incomplete states	35 (73%)	26 (68%)	9 (90%)	0.34	23 (72%)	12 (75%)	0.04
› Mood reactivity and/or fluctuations	42 (88%)	34 (89%)	8 (80%)	0.18	29 (91%)	13 (81%)	0.19
› Atypical depression*	13 (27%)	11 (29%)	2 (20%)	0.12	11 (34%)	2 (13%)	0.31
› Increased appetite	8 (17%)	7 (18%)	1 (10%)	0.17	5 (16%)	3 (19%)	0.05
› Increased weight (≥ 10%)	12 (25%)	9 (24%)	3 (30%)	0.08	7 (22%)	5 (31%)	0.12
› Hypersomnia	20 (42%)	15 (39%)	5 (50%)	0.10	11 (34%)	9 (56%)	0.22

Table 2: Populations' baseline characteristics. QIDS: Quick Inventory of Depressive Symptoms, 16 items – clinician rated version; GAF: Global Assessment of Functioning; SSRI: selective serotonin reuptake inhibitors; SNRI: serotonin and norepinephrine reuptake inhibitors; rTMS: repetitive transcranial magnetic stimulation; ECT: electro-convulsive therapy. Means are expressed in "mean (± standard deviation)"; median are expressed in "median (1st – 3rd quartile)". * According to DSM-5 criteria; ¤ Corresponds to the duration of the episode at the time of the first contact with Strasbourg's expert center on TRD plus the lead-in period, i.e. until the first DATA1 treatment. † The concepts of mixed or incomplete states, mood reactivity or fluctuation are defined according to Karl Leonhard. For tables 2.b and 2.c, Cohen's and Hasselblad-Hedges' *d* are in black bold when the difference is significant (α = 0.05, bilateral, uncorrected for multiple testing), or in dark gray when it is only a trend (threshold = 0.1, bilateral, uncorrected for multiple testing) and in light gray italic otherwise.

Table 4 – Effect size

	4.a – DATA1 (n = 48)			4.b – DATA2 (n = 16)		4.c – Intention to treat (n = 48)					
**Population characteristics**	**Rm**	**nRm**	*d*	**Rm**	**nRm**	**Rm**	**nRm**	*d*	**Rs**	**nRs**	*d*
Population (N)	23 (48%)	25 (52%)		15 (94%)	1 (6%)	37 (77%)	11 (23%)		42 (88%)	6 (13%)	
Female / Male (% female)	10/13 (43%)	12/13 (48%)	0.04	8/7 (53%)	1/0	16/21 (43%)	6/5 (55%)	0.11	18/24 (43%)	4/2 (67%)	0.23
Age (years)	57 ±13	60 ±11	0.25	58 ±12	84	57 ±11	64 ±13	0.60	59 ±11	58 ±13	0.11
Age at first episode (years)	41 ±16	43 ±16	0.15	43 ±20	61	41 ±16	48 ±14	0.47	43 ±17	38 ±12	0.30
Number of previous episodes	2 ±2	2 ±2	0.03	3 ±3	2	2 ±2	1 ±1	0.64	2 ±2	1 ±1	0.66
Bipolar disorder*	5 (22%)	9 (36%)	0.17	5 (33%)	1	10 (27%)	4 (36%)	0.10	11 (26%)	3 (50%)	0.25
Affected first degree relative	16 (70%)	14 (56%)	0.14	9 (60%)	0	25 (68%)	5 (45%)	0.22	27 (64%)	3 (50%)	0.14
Lead-in period (weeks)	16 (7 – 24)	20 (5 – 43)	0.34	9 (4 – 34)	45	14 (5 – 30)	22 (5 – 43)	0.23	13 (5 – 30)	32 (20 – 43)	0.55
**Resistance of current episode**											
Duration of index episode (years)¤	4.5 ±3.1	3.8 ±2.4	0.23	3.7 ±2.5	1.9	3.9 ±2.7	4.5 ±2.2	0.17	3.9 ±2.7	5.8 ±2.4	0.71
Thase and Rush resistance	2.6 ±0.5	3.2 ±0.9	0.80	3.5 ±1	3	2.9 ±0.7	3 ±0.5	0.17	2.9 ±0.7	2.8 ±0.3	0.14
› SSRI	22 (96%)	24 (96%)	0.02	15 (100%)	0	36 (97%)	10 (91%)	0.31	41 (98%)	5 (83%)	0.50
› SNRI	19 (83%)	23 (92%)	0.21	15 (100%)	1	32 (86%)	10 (91%)	0.11	36 (86%)	6 (100%)	–
› Tricyclics antidepressants	13 (57%)	18 (72%)	0.16	9 (60%)	1	23 (62%)	8 (73%)	0.12	26 (62%)	5 (83%)	0.27
› Antipsychotic	10 (43%)	12 (48%)	0.04	10 (67%)	1	18 (49%)	4 (36%)	0.12	18 (43%)	4 (67%)	0.23
› Combination or augmentation	15 (65%)	22 (88%)	0.33	13 (87%)	1	27 (73%)	10 (91%)	0.31	31 (74%)	6 (100%)	–
› rTMS	0 (0%)	4 (16%)	–	2 (13%)	1	2 (5%)	2 (18%)	0.33	2 (5%)	2 (33%)	0.55
› ECT	0 (0%)	6 (24%)	–	5 (33%)	0	5 (14%)	1 (9%)	0.11	6 (14%)	0 (0%)	–
**Clinical features**											
QIDS-C baseline	16.2 ±3.4	17.4 ±3.1	0.38	16.2 ±3.5	17	16.4 ±3.4	16.6 ±3.7	0.27	16.3 ±3.3	17.5 ±4.5	–
GAF baseline	40 ±8	41 ±8	0.08	38 ±9	55	40 ±9	45 ±5	0.60	41 ±8	42 ±6	0.22
› Anxiety	17 (74%)	23 (92%)	0.34	13 (87%)	0	30 (81%)	10 (91%)	0.20	35 (83%)	5 (83%)	0.00
› Psychotic features	3 (13%)	5 (20%)	0.12	4 (27%)	0	8 (22%)	0 (0%)	–	8 (19%)	0 (0%)	–
› Mixed or incomplete states	19 (83%)	16 (64%)	0.24	11 (73%)	1	29 (78%)	6 (55%)	0.24	30 (71%)	5 (83%)	0.17
› Mood reactivity and/or fluctuations	20 (87%)	22 (88%)	0.02	12 (80%)	1	31 (84%)	11 (100%)	–	36 (86%)	6 (100%)	–
› Atypical criteria (DSM-5)	4 (17%)	9 (36%)	0.24	2 (13%)	0	6 (16%)	7 (64%)	0.53	9 (21%)	4 (67%)	0.48
› Increased appetite	5 (22%)	3 (12%)	0.17	3 (20%)	0	7 (19%)	1 (9%)	0.20	7 (17%)	1 (17%)	0.00
› Increased weight (≥ 10%)	5 (22%)	7 (28%)	0.08	5 (33%)	0	10 (27%)	2 (18%)	0.15	11 (26%)	1 (17%)	0.14
› Hypersomnia	8 (35%)	12 (48%)	0.13	9 (60%)	0	15 (41%)	5 (45%)	0.06	16 (38%)	4 (67%)	0.28

**Treatment**												
Patients on lithium - n (%)	10 (43%)	9 (36%)	*0.07*	6 (40%)	1 (100%)	16/35 (46%)*	1/11 (9%)*	0.51	16/40 (40%)*	1/6 (17%)*	*0.29*
MAOI (mg/d of TCP-eq)	49 ±19	49 ±18	*0.03*	52 ±16	50	52 ±18	40 ±18	*0.62*	51 ±17	36 ±23	*0.87*
D2RAG (mg/d of PPX-eq)	1.9 ±0	4.7 ±2.2	1.74	3.2 ±2.5	1	3.1 ±2.3	1.8 ±1.1	*0.60*	3 ±2.3	2 ±1.3	*0.48*
**Results**												
QIDS-C (end | final)	2.7 ±1.8	11.3 ±4	**2.74**	2.2 ±1.4	8	2.5 ±1.7	10.5 ±4.6	**2.99**	2.9 ±1.8	14.5 ±4.7	**5.08**
Time to Rm | trial completion (weeks)	11 (7 – 15)	4 (3 – 26)	*0.72*	12 (4 – 25)	–	14 (6 – 93)	142 (27 – 294)	**1.41**	16 (6 – 93)	153 (138 – 294)	**1.79**
GAF final	75 ±10	–	–	72 ±13	30	74 ±11	57 ±16	**1.40**	74 ±10	44 ±8	**3.02**

Table 4: Predictors of Rm and Rs with DATA. **4.a**. Difference between remitters (Rm) and non-remitters (nRm) after DATA1. **4.b**. Difference between Rm and nRm after DATA2. **4.c.** ITT (intention to treat analysis), i.e. after DATA considered as a whole. Left columns: Difference between Rm and nRm. Right columns: Difference between responders (Rs) and non-responders (nRs). * During the second phase, lithium was discontinued and replaced by lamotrigine in two patients who are excluded from this analysis (potential bias). Cohen's and Hasselblad-Hedges' *d* are in black bold when the difference is significant (α = 0.05, bilateral, uncorrected for multiple testing), or in dark gray when it is only a trend (threshold = 0.1, bilateral, uncorrected for multiple testing) and in light gray italic otherwise.

Time to relapse – comparison between studies

Methods

Data were extracted from two publications:

1. Figure 2 from Fekadu et al. (2012) (3). Unfortunately the latter separated survival curves between low or average social support *vs* strong social support without specifying the repartition between groups and remaining proportion of patients. Moreover the curves were adjusted for years of education, gender, age at onset, discharge clinical status and diagnosis (unipolar, bipolar, secondary). The only data provided were the initial number of responders (Rs, n = 95) and the final number of (known) relapses (n = 52). Interpreting each step as 1 or 2 relapses depending on the size of gap, we could simulate the most likely proportion of patients in the two groups to 'average' the two curves (strong support, n = 51 ; low or average, n = 44) assuming attrition to be exponential attrition.
2. Figure 2 from Rush et al. (2016) for the STAR*D cohort (4). Numbers were specified so that results from step 3 and 4 could be combined unambiguously.

Results

Population characteristics

Population characteristics and of the different cohorts are summarized in the following table.

	Fekadu et al. 2012	Rush et al. 2016 (STAR*D)	CADOT
**Recruitment**	Initial inpatient treatment<br>Tertiary care<br>UK	Outpatients<br>Primary + secondary care, USA<br>Step 3, 4, 5	Outpatients with TRAD<br>Quaternary care<br>France
**Age**	48 ±12	44 ±12	59 ±12
**Gender ratio**	2.9	1.0	0.8
**Episode duration**	5.5	2.8	4.1
**Atypical**	–	21%	27%
**Bipolar**	23%	*No mania, hypomania, mixed phase*	29%
**Rm**	60%	13%	77%
**Rs**	80%	30%	88%
**N follow-up**	95	151	42

Rm: remitters. **Rs:** responders (including remitters). **TRAD**: Treatment resistant anergic-anhedonic depression.

Remark: Fekadu et al. report that 16% of the patients received MAOI, and that these patients were more likely to reach remission (Rm), particularly for unipolar treatment-resistant depression (odds ratio (OR) = 6.5) and to remain in remission at the end of the follow-up (OR = 4.8). However, this includes an unknown proportion of patients taking moclobemide which only inhibits MAO_A and hence has no effect on dopaminergic tonus.

Survival curves

In the following figure, the survival curve of CADOT (blue) is compared to the one of STAR*D (step 3 and 4, gray) and of a cohort followed after inpatient treatment in an expert center for treatment resistant depression (TRD – expert, green). Numbers give the proportion of patients at risk relative to the initial sample.

Importantly 'TRD – expert' curve and numbers could only be estimated and may slightly differ from the real ones (see methods).

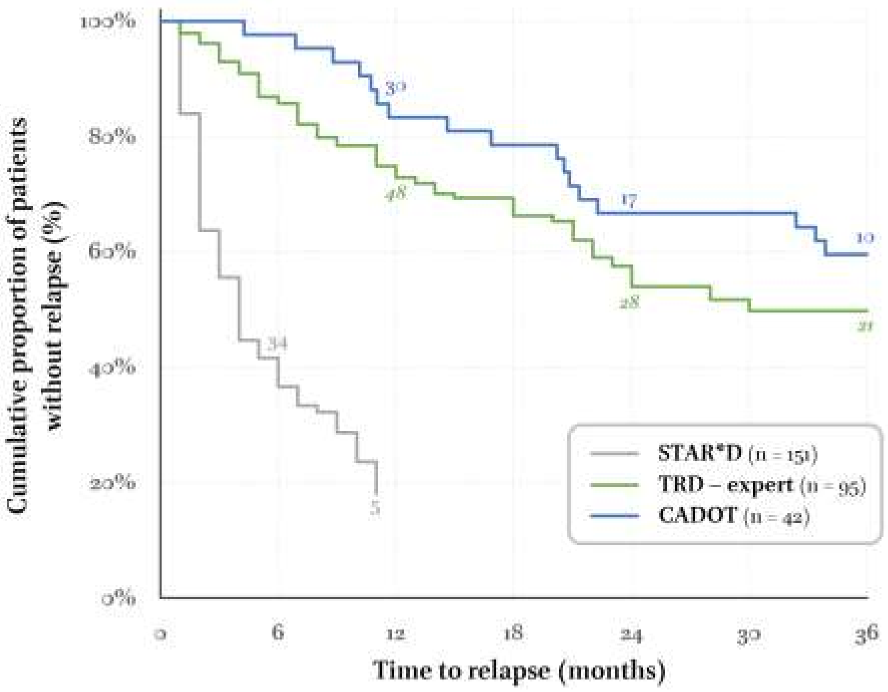

Personality traits in CADOT

Personality disorders and other comorbidities have been analyzed at the level of the full sample of TRAD (n = 61). Since this report is in French (submitted*), we provide an English digest limited to the 48 patients included in CADOT.

Methods

We did not use structured interview instruments for the diagnosis of personality disorders, and these were not considered to be exclusion criteria in CADOT.

On follow up, personality traits that have been established by consensus between at least two clinicians (mostly JF and OM). This was performed in patients who have been followed over an extended period (>1.5 years) during which most (but not all) have shown notable and lasting improvement, up to remission.

Results

At inclusion

Personality disorders were screened but could not account for the full TRAD clinical picture. None of the patient fulfilled any DSM personality disorders when proposed with DATA (especially no patient with borderline personality disorders).

One patient fulfilled Stein's '*hysteroid dysphoria*' clinical picture (the one which precedes the New-York school version of 'atypicality' endorsed in the DSM) (5). If emotional hyper-expressivity and seductive behavior were still perceptible once in remission, the patient was far from fulfilling the diagnosis of histrionic personality disorder.

Residual personality during the follow-up

Personality traits persisted or appeared during the improvement / remission in 9 patients. Traits mainly belonged to cluster C (n = 6), less often to cluster B (histrionic, n = 1; narcissistic, n = 2). In all cases, however, traits were isolated: no patient met the criteria for either personality disorder.

Interestingly, all but one of these patients met the criteria for 'atypicality' during the episode (8/9) which is in line with the original meaning of the inventors of the label (West and Dally, St Thomas's hospital in London) (6).

Patients with significant traits corresponded to the majority of Rs/nRm (responders/non-responders = 4/5) and to nRs (non-responders = 3/5) who did not want to enter DATA2 after partial or complete failure of DATA1. This might be another way to account for the poorer outcome in patients fulfilling 'atypical' features (ITT analysis for Rm/nRm or Rs/nRs – table 4.c).

Last, one of the two patients with noticeable personality trait who had a complete remission after MAOI alone, was the '*hysteroid dysphoria*' patient (consistent with Liebowitz and Stein's original hypothesis) (5).

* "*De la dépression au syndrome anergique-anhédonique dopa-sensible – Psychopathologie différenciée et neuropsychiatrie de précision – réanalyse d'une série de cas*" (From TRAD to anergic-anhedonic dopa-sensitive syndrome –reanalysis of a case series) Dormegny-Jeanjean et al. (Foucher JR for correspondence).

Remark

It is difficult to draw any conclusion from these retrospective observations. Thought traits were only considered if already present before the episode, many of these patients (7/9) did not reach full remission. This is likely biasing our assessment, not to mention the difficulty of separating what belongs to the depressive state and to the personality, traits of which may have been accentuated after such a prolonged depressive episode (4.1 years).

Bibliography

1. Cohen J. *Statistical power analysis for the behavioral sciences*. 2nd ed. New York, NY (USA): Routledge (1988). doi:10.4324/9780203771587

2. Hasselblad V, Hedges L V. Meta-analysis of screening and diagnostic tests. *Psychol Bull* (1995) **117**:167–178. doi:10.1037/0033-2909.117.1.167

3. Fekadu A, Rane LJ, Wooderson SC, Markopoulou K, Poon L, Cleare AJ. Prediction of longer-term outcome of treatment-resistant depression in tertiary care. *Br J Psychiatry* (2012) **201**:369–375. doi:10.1192/bjp.bp.111.102665

4. Rush AJ, Trivedi MH, Wisniewski SR, Nierenberg AA, Stewart JW, Warden D, Niederehe G, Thase ME, Lavori PW, Lebowitz BD, et al. Acute and longer-term outcomes in depressed outpatients requiring one or several treatment steps: a STAR*D report. *Am J Psychiatry* (2006) **163**:1905–1917. doi:10.1176/ajp.2006.163.11.1905

5. Liebowitz MR, Klein DF. Hysteroid dysphoria. *Psychiatr Clin North Am* (1979) **2**:555–575. doi:https://doi.org/10.1016/S0193-953X(18)30996-1

6. West ED, Dally PJ. Effects of iproniazid in depressive syndromes. *Br Med J* (1959) **1**:1491–1494. doi:10.1136/bmj.1.5136.1491
